# Comparison of extracellular vesicle isolation and storage methods using high-sensitivity flow cytometry

**DOI:** 10.1371/journal.pone.0245835

**Published:** 2021-02-04

**Authors:** Sarah Deville, Pascale Berckmans, Rebekka Van Hoof, Ivo Lambrichts, Anna Salvati, Inge Nelissen

**Affiliations:** 1 Health Unit, Flemish Institute for Technological Research, Mol, Belgium; 2 Biomedical Research Institute, Hasselt University, Diepenbeek, Belgium; 3 Theoretical Physics, Hasselt University, Diepenbeek, Belgium; 4 Laboratory for Soft Matter and Biophysics, KU Leuven, Leuven, Belgium; 5 Groningen Research Institute of Pharmacy, University of Groningen, Groningen, The Netherlands; The Ohio State University, UNITED STATES

## Abstract

Extracellular vesicles (EVs) are of interest for a wide variety of biomedical applications. A major limitation for the clinical use of EVs is the lack of standardized methods for the fast and reproducible separation and subsequent detection of EV subpopulations from biofluids, as well as their storage. To advance this application area, fluorescence-based characterization technologies with single-EV resolution, such as high-sensitivity flow cytometry (HS-FCM), are powerful to allow assessment of EV fractionation methods and storage conditions. Furthermore, the use of HS-FCM and fluorescent labeling of EV subsets is expanding due to the potential of high-throughput, multiplex analysis, but requires further method development to enhance the reproducibility of measurements. In this study, we have applied HS-FCM measurements next to standard EV characterization techniques, including nanoparticle tracking analysis, to compare the yield and purity of EV fractions obtained from lipopolysaccharide-stimulated monocytic THP-1 cells by two EV isolation methods, differential centrifugation followed by ultracentrifugation and the exoEasy membrane affinity spin column purification. We observed differences in EV yield and purity. In addition, we have investigated the influence of EV storage at 4°C or -80°C for up to one month on the EV concentration and the stability of EV-associated fluorescent labels. The concentration of the in vitro cell derived EV fractions was shown to remain stable under the tested storage conditions, however, the fluorescence intensity of labeled EV stored at 4°C started to decline within one day.

## Introduction

Extracellular vesicles (EVs) are lipid-based membrane-bound vesicles of different sizes and content, which are released by cells either constitutively or in a regulated manner. EVs carry a specific subset of proteins, nucleic acids, lipids and metabolites which reflect both the cell type and mechanism of origin. The release and subsequent uptake of EVs represents a biologically significant communication system. EVs transfer their content to recipient cells, and the transfer of the molecular and genetic cargo is accompanied by the reprogramming of the recipient cell functions [1]. The number of EVs and composition of EVs`cargoes are highly heterogenous and dynamic, and dependent on the cell origin, activation status and environmental conditions. Several studies have shown that EVs and disturbed EV communication mechanisms are implicated in multiple disease processes, including cancer and metastatic spread, cardiovascular diseases and neurological disorders [2]. Therefore, the understanding of the role of EV cargo molecules and their distribution within the body will open new opportunities for a wide variety of biomedical applications, such as the early monitoring of diseases, but also as primary therapeutics and drug delivery vehicles [3].

Multiple methods are available for the separation and enrichment of EVs from different biofluids. Many methods utilize specific physical EV properties, i.e. their small size and low density. These include ultracentrifugation [4], size exclusion chromatography [5], density gradient centrifugation [6] and asymmetric-flow field-flow fractionation [7], among others. Other methods are based on precipitation of the EVs by—for instance—polyethylene glycol [8] or protamine [9]. More advanced methods are based on biochemical affinity [10, 11] which can be combined with microfluidic technologies [12]. The different stand-alone or combined methods all have their advantages and shortcomings, resulting in different EV yield and purity which influence further downstream analysis [4, 13]. Therefore, an elaborate physicochemical characterization of the isolated EV subsets is strongly encouraged to demonstrate the nature of the isolated subsets and to allow comparison of results [14, 15]. Current workflows for the separation of EVs are time-consuming and often impose the need for intermediate storage of EV samples, which introduces an additional factor of uncertainty regarding the stability of EV fractions over time. Earlier investigations have reported inconsistencies about the effect of EV storage on biophysical and functional properties [16–20], indicating that storage protocols will most likely need to be tuned to the applied EV isolation procedures and read-out parameters. For the characterization of EVs’ concentration, size and biomarker distribution various technologies are available including electron microscopy, nanoparticle tracking analysis (NTA), dynamic light scattering, tunable resistive pulse sensing and high-sensitivity flow cytometry (HS-FCM) [14]. Among these, HS-FCM is emerging as a promising technology to evaluate the quantity and the phenotype of EVs with high-throughput and multiplex fluorescence possibilities [13, 21]. Flow cytometers can detect EVs based on their light scattering properties or—after labelling with fluorescent molecules—fluorescence intensity at the single-EV level, thereby distinguishing themselves from NTA which is based on similar detection principles but provides bulk EV measurements. Due to their small size, however, EVs are weak light scatterers, in the same range as the instrument’s electronic noise and/or biomolecules that co-purify from biological samples, thus limiting their adequate detection [22]. Recent work has demonstrated that the implementation of violet side scatter detection can help to improve the measurement of smaller EVs compared to traditional scatter detection [23]. Alternatively, triggering on fluorescence intensity of labelled EVs has been proposed because it has the advantage of improved signal detection separated from background noise [24]. In addition, well-established calibration methods have become available and reference particles including reference EVs are under development for comparison of scatter and fluorescence read-out between different instruments and labs [25–29]. Although the use of HS-FCM is steadily expanding, there is still a high need for method validation to enhance the reproducibility of measurements, including development of robust protocols, workflows and quality controls [30].

In this work, we have compared the yield and purity of EV fractions separated from lipopolysaccharide (LPS)-stimulated monocytic THP-1 cells by two distinct methods: differential centrifugation combined with ultracentrifugation (DC + UC) and the affinity spin column-based exoEasy separation. *In vitro* stimulated cell cultures were selected as a readily available, standardized and productive source of EVs. The EV separation methods were selected as two technically distinct methods, with DC+UC being widely used as a golden standard method for research purposes and resulting in highly concentrated samples in physiological buffer, while exoEasy being a commercial affinity column-based method which is much faster and results in EVs concentrated in a proprietary elution buffer. EVs were analyzed using diverse techniques, including scatter-based nanoparticle tracking analysis (NTA) and fluorescence-triggered HS-FCM after staining of EVs with 5-(and-6)-Carboxyfluorescein Diacetate Succinimidyl Ester (CFDA-SE). To gain further insight in the importance of the EV isolation method, the effect of storage at 4°C or -80°C on EV concentration and the fluorescence stability of CFDA-SE-labeled EVs were studied.

## Materials and methods

### Cell culture

THP-1 cells (ATCC, TIB-202, Manassas, USA) were grown in RMPI 1640 medium with GlutaMax (Gibco, Grant Island, USA, #11554526) supplemented with 10% fetal bovine serum (FBS) (Biochrom, Berlin, Germany, #S0615). Cell numbers were maintained between 200 000 and 800 000 cells/ml. For routine subculturing and cell viability assessment as a quality control, THP-1 cells were counted by a NucleoCounter NC-100 (ChemoMetec, Lincoln, USA) and cultures were split every 3–4 days using fresh cell culture medium.

### Extracellular vesicle production

For EV production and harvest, THP-1 cells were collected in 50 ml polypropylene tubes (Greiner Bio One, Frickenhausen, Germany, #227261) by 10 minutes of centrifugation at 200 rcf and 20°C in a swinging bucket Rotanta 460 R centrifuge (Hettich, Tuttlingen, Germany). Next, they were seeded at a density of 1×10^6^ cells/ml in a T75 cm^2^ tissue culture treated cell culture flask (Greiner Bio One, #658170) in 15 ml of RPMI 1460 medium with GlutaMAX supplemented with 2% EV-depleted FBS (Gibco, #A2720801), and stimulated with 100 ng/ml lipopolysaccharide (LPS) from *E*. *coli* serotype 055:B5 (Sigma-Aldrich, St. Louis, USA, #L2880). After 24 hours of incubation at 37°C, 5% CO_2_ and 95% humidity, cells and the conditioned cell medium were collected.

### Extracellular vesicle isolation and storage

For the DC + UC separation procedure, the conditioned cell medium was first purified in order to remove cells and cellular debris by means of differential centrifugation. The conditioned cell medium was centrifuged in 15 ml and 50 ml polypropylene tubes (Greiner Bio One, #188271 and #227261) at 4°C with 10 minutes steps at 300 rcf, 400 rcf, 500 rcf and 500 rcf in a swinging bucket Rotanta 460 R centrifuge with maximal brake. Next, medium was centrifuged at 10,000 rcf for 30 minutes at 4°C to remove larger particles and 100,000 rcf for 65 minutes at 4°C in order to pellet the EVs, both using 17 ml thinwall polypropylene tubes (Beckman Coulter, Brea, USA, #103277) in an Optima XPN-80 ultracentrifuge with a swinging bucket SW32.1 Ti rotor (Beckman Coulter, *k-*force: 3571 and 357.1) at maximal brake. The EVs from 15 ml of conditioned cell medium were resuspended in 30 μl of PBS without Ca^2+^ and Mg^2+^ which was filtered over a 0.1 μm cut-off filter (Merck Millipore, Tullagreen, Ireland, #SLV033RS) and the EVs were transferred to LoBind Eppendorf tubes (Eppendorf, Hamburg, Germany, #022431081) for further downstream analyses. For the exoEasy separation procedure, the conditioned cell medium was first purified in order to remove cells and cellular debris by centrifugation using and 50 ml polypropylene tubes (Greiner Bio One, #227261) at 300 rcf, and subsequently the medium was filtered over a 0.8 μm cut-off filter (Merck Millipore, #SLAA0033SB). Thereafter, the exoEasy protocol (Qiagen, Hilden, Germany) was proceeded according to the manufacturer’s guidelines. The EVs were eluted in 500 μl of exoEasy elution buffer and transferred to LoBind Eppendorf tubes. In order to compare the results between different separation methods and biological replicates, the same start volume was used, and all downstream analyses are normalized to the equivalent of EVs obtained from 1×10^6^ LPS-stimulated THP-1 cells. Equal volumes of blank cell culture medium containing 2% EV-depleted FBS (Gibco) and 100 ng/ml LPS (‘blank medium’) were processed in parallel to the EV samples using the aforementioned methods to serve as background control for the downstream measurements.

To evaluate the effect of storage on the unlabeled EVs, sample replicates (n = 3) were aliquoted in LoBind Eppendorf tubes. Aliquots were transferred to a fridge for storage at 4°C or a freezer for storage at -80°C. For the storage of the unlabeled EVs, two independent DC + UC and exoEasy EV isolations were evaluated immediately after EV purification or after 1 week, 2 weeks and 1 month of storage. The first, samples were thawed at room temperature until completely defrosted (approximately 30 minutes). Thereafter, NTA and HS-FCM were performed. For the storage of the labeled EVs, freshly isolated EVs from two independent DC + UC and exoEasy EV isolations were stained with CFDA-SE and purified using sucrose density gradient centrifugation. Thereafter, sample replicates (n = 3) were aliquoted in LoBind Eppendorf tubes and stored in a fridge at 4°C or transferred to a freezer at -80°C. After 1 day, 5 days, 7 days, 2 weeks, 3 weeks and 4 weeks, the samples were thawed at room temperature until completely defrosted (approximately 30 minutes). Subsequently, HS-FCM was proceeded.

### Protein assay

Protein concentrations of the freshly isolated EV fractions were determined by the Micro BCA^™^ Protein Assay Reagent Kit (ThermoFisher Scientific, Waltham, USA) at 37°C according to the manufacturer’s guidelines. The absorbance was read out at 561 nm (Clariostar, BMG Labtech, Offenburg, Germany). A standard curve of serially diluted Bovine Serum Albumin (ThermoFisher Scientific) in 0.1 μm filtered PBS was used.

### Nanoparticle tracking analysis

EV size and concentrations were determined by means of nanoparticle tracking analysis (NTA) using a NanoSight NS500 instrument (NanoSight, Wiltshire, UK) equipped with a green 532 nm laser (max power < 80 mW) and a sCMOS camera, and NTA software version 3.0 according to the manufacturers’ guidelines. Samples were diluted in 0.1 μm filtered PBS prior to loading into the sample chamber. For every sample, 3 videos of 60 seconds were recorded (1499 frames with 25 frames/second) with a camera level set at 13. For the analysis, a detection threshold of 7 was used. The number of particles/frame ranged from 40 to 90. Particle sizes are reported as mode of a particle number concentration-based size distribution.

### Western blotting

Normalized volumes of the EV fractions corresponding to equal amounts of the starting volume of the conditioned cell culture medium and background controls were heated at 95°C in Laemmli sample buffer containing a final concentration of 5% SDS, 15 mM Tris, 0.025% glycerol and 1.25% β-mercaptoethanol (Bio-Rad). Samples were subjected to gel electrophoresis on a 4–20% mini PROTEAN SFX (Bio-Rad) at 130 V for 1 hour. SDS-PAGE gels were blotted on a nitrocellulose membrane using the Trans-Blot^®^ Turbo^™^ RTA Mini Nitrocellulose Transfer Kit and Blotting System (Bio-Rad). Thereafter, membranes were blocked in 5% non-fat dry milk (Bio-Rad) in PBS containing 0,1% Tween^®^20 (PBS-T, Sigma-Aldrich) for 2 hours with gentle shaking at room temperature. Next, the primary antibodies for detection of TSG101 (1/500, Santa Cruz Biotechnology, Dallas, USA, #sc-7964), Flottilin-1 (1/500, Santa Cruz Biotechnology, #sc-133153), CD9 (1/500, Santa Cruz Biotechnology, #sc-13118) and cytochrome C (1/500, Santa Cruz Biotechnology, #sc-13156) were added overnight at 4°C in solution 1 from the SignalBoost^™^Immunoreaction Enhancer Kit (Merck MilliPore). After three washing steps of 5 minutes each with PBS-T, an HRP-conjugated donkey anti-mouse IgG (1/5000, Jackson Immuno Research, West Grove, USA, #715-035-151) was added for 2 hours at room temperature in solution 2 from the SignalBoost^™^Immunoreaction Enhancer Kit. Thereafter, membranes were washed and developed using the Clarity Western ECL Substrate kit (Bio-Rad) according the manufacturer’s guidelines. Membranes were imaged using a ChemiDoc XRS+ System (Bio-Rad).

### Flow cytometry

Prior to analysis, EV isolates and parallel blank medium samples were fluorescently stained with 10 μM Vybrant^™^ CFDA-SE (ThermoFisher Scientific, #V12883) as previously described [31, 32]. Stained EVs were further purified overnight by bottom-up density gradient centrifugation using either an iodixanol gradient (Figs 3 and 4) or a sucrose density gradient (Figs 5 and 6). In order to prepare a bottom-up iodixanol gradient, samples with a final volume of 300 μl were mixed with 1 ml of 60% iodixanol (Optiprep, StemCELL Technologies, Cologne, Germany, #07820). This mixture was overlaid with 700 μl of 40% iodixanol, 700 μl of 30% iodixanol and 2 ml of 10% iodixanol. Iodixanol dilutions were prepared by dilution of the 60% iodixanol to 40% iodixanol using a homogenization buffer containing 6 mM EDTA, 60 mM Tris-HCl and 0.25 mM sucrose (pH 7.4) and subsequent dilutions were performed by dilution of 40% iodixanol to 30% iodixanol and 10% iodixanol using a homogenization buffer containing 1 mM EDTA, 10 mM Tris-HCl and 0.25 mM sucrose (pH 7.4). Samples were centrifuged for 14 hours at 367,600 rcf and 4°C in 5 ml open-top thinwall polyallomer tubes (Beckman Coulter, #103242) using an Optima XPN-80 ultracentrifuge and a SW55 Ti rotor (Beckman Coulter, k-factor: 48.5) with moderate acceleration/braking (5/5). Fractions of 480 μl were collected and measured. The density of the iodixanol fractions was determined by absorbance spectroscopy at 340 nm (Clariostar, BMG Labtech, Ortenberg, Germany) according to the manufacturer’s recommendations. Briefly, different 1:1 aqueous dilutions of iodixanol solutions (5–40%) were prepared and the absorbance was determined. Using this standard curve, the density of the fractions collected from a control iodixanol gradient was calculated.

In order to prepare a bottom-up sucrose density gradient, the procedure described by *van der Vlist et al* [24] was followed and samples were centrifuged for 16 hours at 187,600 rcf and 4°C in 17 ml thinwall polypropylene tubes (Beckman Coulter, #103277) using an Optima XPN-80 ultracentrifuge (*k*-force: 229.4) with swinging bucket SW32.1 Ti rotor with acceleration/moderate braking (5/5). Sucrose fractions of 1 ml were collected in LoBind Eppendorf tubes and either analyzed directly or stored at 4°C or -80°C before measurement.

A BD Influx flow cytometer equipped with a high power 488-nm laser (200 mW) and a small-particle detector for high sensitivity forward scatter detection (Becton Dickinson, Erembodegem, Belgium) was used for analysis. The device utilizes a highly sensitive fluorescence trigger to measure the EVs as described before by *van der Vlist et al*. [24]. The threshold of the FL-1 fluorescent channel (BP530/40) was set to 0.30 arbitrary units, which corresponds to 25 units of FITC MESF. The gain of the forward scatter detector was set to 47.74, the gain of the side scatter detector was set to 52.28 and the gain of the FL-1 fluorescent channel (BP530/40) was set to 49.96. Samples were measured at a flow rate of approximately 10 μl/minute (event rate < 4000 events/s) and run for 30 seconds to determine their event count and calculate their concentration. For the monitoring of EV concentrations over time, the precise sample flow rate was calculated at the beginning and end of every experiment by weighting a tube filled with distilled water before and after a 10 minute run of sample. EV concentrations were normalized by the calculated flow rate during each experiment to allow comparison of EV concentrations between experiments, Yellow-Green fluorescent beads of 100 and 200 nm (ThermoFisher Scientific, #F8803 and F8811) were used for validation of laser alignment in small particle detection mode. Conversion to units of FITC MESF was performed using FITC MESF Beads (custom product manufactured by Becton Dickinson Biosciences, 2 μm FITC Quantitation Beads; R&D Lot #2352–87) corresponding to 98 704 (bead 1), 20 292 (bead 2), 10 552 (bead 3) and 4 315 (bead 4) units of FITC MESF (S1 Fig in S1 File). Before measuring, 0.5 ml PBS supplemented with 0.5% BSA (Sigma-Aldrich) was added to the FITC MESF Beads to get a concentration of 2 × 10^4^ of each population/tube. The beads were measured by triggering on forward scatter detector with a threshold of 0.30, the gain of the forward scatter detector was set to 9.20, the gain of the side scatter detector was set to 17.50 and the gain of the FL-1 fluorescent channel (BP530/40) was set to 49.96. A sheath pressure of 4.9 and sample pressure of 5.9 was used to measure 10,000 events at an event rate of 300. The FL-1 fluorescent channel intensities of the different bead populations were determined. A calibration curve was prepared, and the flow cytometric data were converted to units of FITC MESF using FlowJo 10.7.1 software (Becton Dickinson). The brightness of the 100 and 200 nm beads corresponds to 121 and 872 units of FITC MESF, respectively. In order to confirm that our EV analyses were robust, not suffering from swarming effects in the operational range, samples were serially diluted in 0.1 μm filtered PBS and measured. The absence of swarming was confirmed by showing proportional increase of the event rate with increasing concentration of EVs.

To evaluate the true nature of EVs in our analyses, in contrast to non-vesicular contaminants, detergent control measurements were performed. For this, isolated EVs and blank medium control samples were stained using the Vybrant^™^ CFDA-SE Cell Tracer Kit (*vide supra*), supplemented with 0.5% (w/v) Triton X-100 (Sigma-Aldrich) or 0.5% (w/v) SDS (Sigma-Aldrich), sonicated in a water bath for 30 minutes, and subsequently incubated in dark at room temperature for 30 minutes. As per the CFDA-SE staining protocol, the samples were further purified by bottom-up density gradient centrifugation using an iodixanol gradient. Flow cytometric data were analyzed using FlowJo 10.7.1 software. Flow cytometry measurements are deposited in the FlowRepository database (FR-FCM-Z375).

### Transmission electron microscopy

EVs were examined by transmission electron microscopy (TEM) imaging. Three droplets of the EV isolated using DC + UC or exoEasy were placed on a clean Parafilm. Afterwards, a carbon coated TEM grid was placed on top of the droplets and allowed to stand for 60 min to adsorb the fluid. The grid with adherent EVs was washed for 3 times with 0.22 μm filtered PBS for 2 min, and 5 times with 0.22 μm filtered ultrapure water for 2 min. Next, EVs were fixed with 2% glutaraldehyde for 10 min, and then washed 5 times with filtered water for 2 min. Grids were transferred to 2% uranyl acetate and allowed to stand for 15 min. Thereafter, grids were incubated in 0.13% methyl cellulose (K5-8) and 0.4% uranyl acetate for 10 min. Finally, grids were dried at room temperature before examination with Tecnai G2 Spirit BioTWIN (FEI, Oregon, USA).

### Statistical analyses

Statistical analyses shown in Fig 4B were performed by GraphPad Prism version 5.00 (GraphPad Prism version 5.00 for Windows, San Diego, California, USA) using the built-in analysis tool and an unpaired, two-tailed Student’s t-test was carefully chosen. Relative EV numbers (%) of EVs with and without detergent treatment were determined using HS-FCM. The results are presented as the average percentage ± standard deviation of three biological replicates. For every biological replicate, the average percentage was determined based on three technical replicate measurements. Results were considered significantly altered when p < 0.05. Statistically significant results are indicated by an asterisk (* p-value < 0.01, ** p-value < 0.001).

### EV-TRACK

We have submitted all relevant data of our experiments to the EV-TRACK knowledgebase (EV-TRACK ID: EV190046) [33].

## Results and discussion

### Isolation by ultracentrifugation versus exoEasy affinity column yield distinct EV subsets with different purity

**Fig 1:**
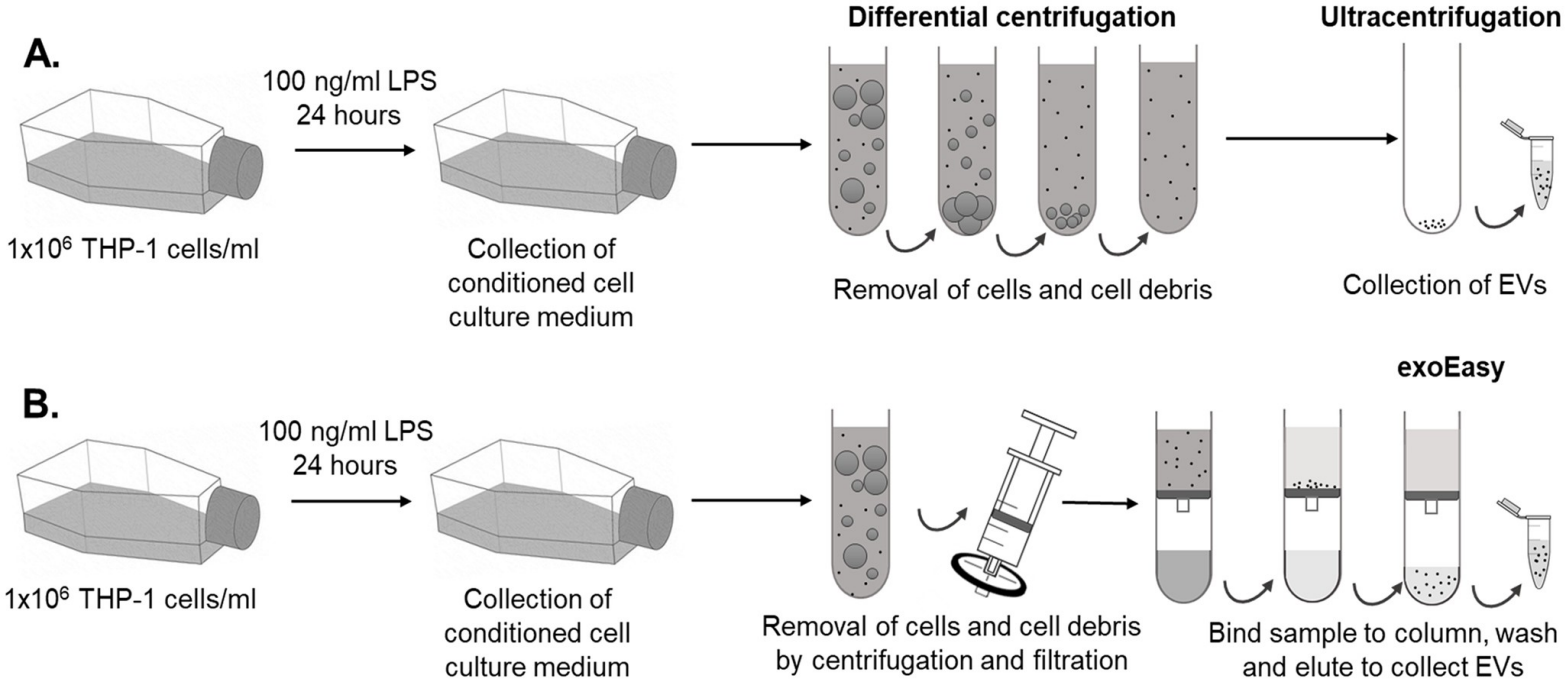
Schematic overview of the EV separation procedures used. (A) differential centrifugation and ultracentrifugation and (B) exoEasy purification. THP-1 cells were seeded at a density of 1 million cells/ml and exposed to 100 ng/ml LPS for 24 hours in medium containing 2% exosome-depleted FBS. Thereafter, the conditioned cell medium containing the EVs was further processed as described in Materials and Methods.

**Fig 2:**
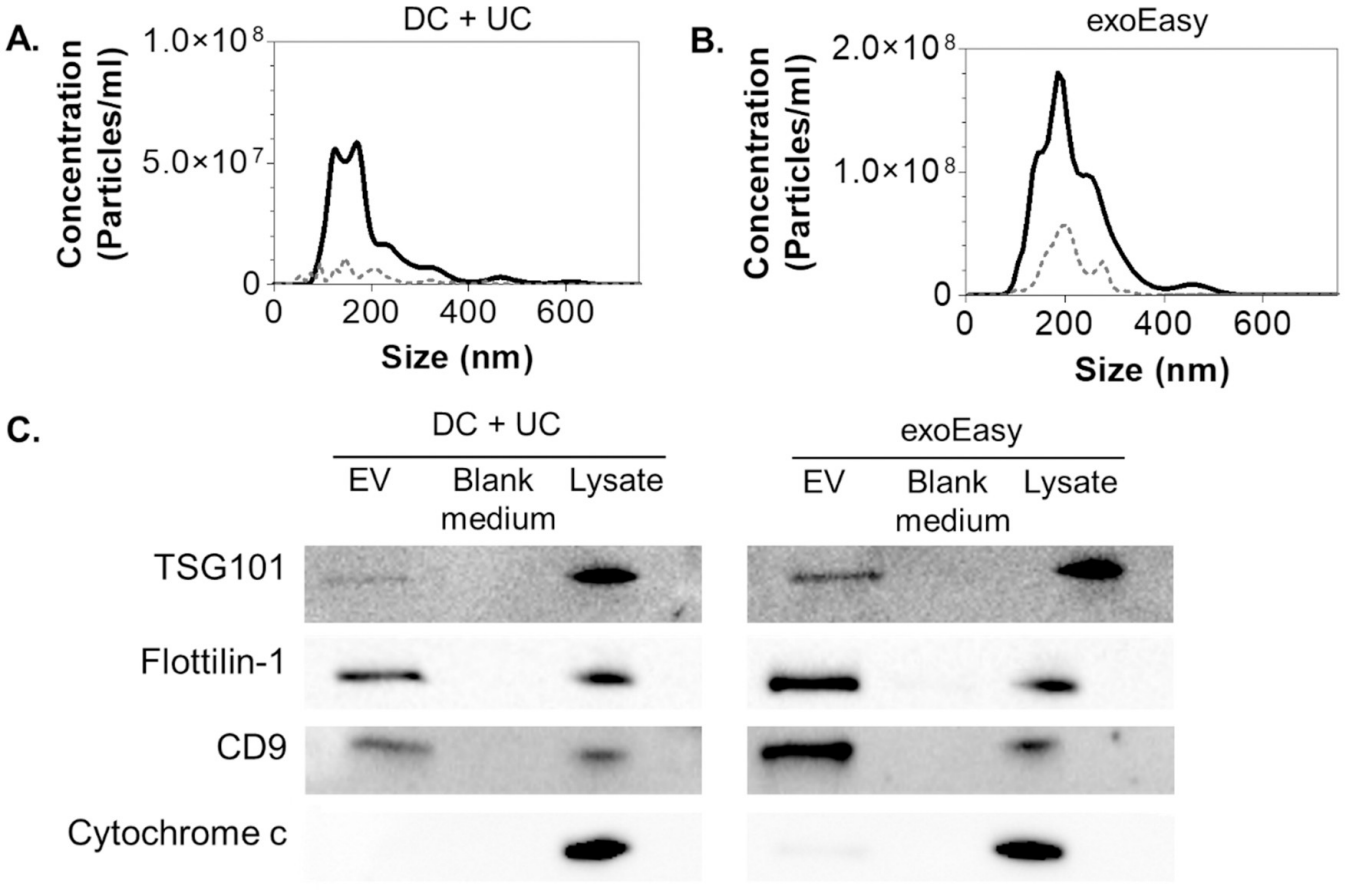
Characterization of EVs by scatter-based NTA and protein analysis of EVs. A representative size distribution profile of particles (solid line) is shown together with the blank medium as background control (dashed line) for **(A)** DC + UC and **(B)** exoEasy. Normalized values are shown to the equivalent of EVs isolated from 1 × 10^6^ LPS-stimulated THP-1 cells as described in Materials and Methods. **(C)** Western blotting analysis of proteins considered as EV-specific markers (TSG101, flottilin-1 and CD9) and the non-EV marker cytochrome c for both separation procedures. The presence or absence of the protein markers in isolated EV fractions was evaluated against blank medium (negative control) and LPS-stimulated THP-1 cell lysate (positive control). Full images of immunoblots are shown in S3 Fig in S1 File.

Extracellular vesicles from the conditioned medium of LPS-stimulated THP-1 cell cultures were separated using (i) differential centrifugation and ultracentrifugation (DC + UC) and (ii) the exoEasy affinity spin column-based purification method (Fig 1). Scatter-based NTA was performed to determine the EVs’ size distributions and quantify their concentrations (Fig 2A and 2B, Table 1). Both separation procedures resulted in EV populations with a heterogeneous size distribution, with a diameter ranging from 75 to 600 nm. The mean size was 182 ± 8 nm for EVs isolated with DC + UC and 210 ± 11 nm for exoEasy. The concentration of detected particles derived from 1 × 10^6^ THP-1 cells was approximately four times higher when samples were isolated using the exoEasy kit, as compared to DC + UC, whereas the protein concentration in the samples was comparable between both separation methods. As a result, when comparing the particle-to-protein ratio [34], the exoEasy kit resulted in higher levels compared to DC+UC (Table 1), which suggests either a more pure EV separation using exoEasy kit or, alternatively, the co-elution of particulate structures from the cell culture medium and/or the spin column which are detected by scatter-based NTA. The latter was found to be most likely since a high particle count was measured in the exoEasy purified blank medium using NTA which were mainly non-proteinous in nature (Fig 2B, Table 1), as well as in the flow-through of blank elution buffer (8.5 × 10^9^ particles/ml) (S2A Fig in S1 File) [10]. The composition of the samples was further explored by Western Blot. A set of EV-specific markers including TSG101, flottilin-1 and CD9 was investigated, while the absence of mitochondrial cytochrome c was used as a negative control (Fig 2C). For all EV-associated markers, the signals from the exoEasy kit were higher compared to DC + UC. In addition, a faint band of cytochrome c was identified in the exoEasy isolates indicating the co-enrichment of mitochondrial components. All blank medium samples were negative for the investigated markers. Electron microscopy imaging of EVs isolated with DC + UC showed typical donut-shaped vesicles, which were heterogenous in size (S4 Fig in S1 File). In the samples separated using exoEasy, no EVs were observed. This is in accordance with previous research which indicated that exoEasy elution buffers generated strong background in electron microscopy [35].

**Table 1:** Characterization of the EVs isolated from THP-1 cells using DC + UC and the exoEasy kit.

		Mean size (nm)	Concentration (× 10^8^ particles/ml)	Protein concentration (μg/ml)	Particles to protein ratio (× 10^8^ particles/μg)
DC + UC	EVs	182 ± 8	8.5 ± 1.0	2.87 ± 0.98	3.2 ± 0.7
	blank medium	149 ± 5	0.2 ± 0.1	0.31 ± 0.01	0.6 ± 0.2
exoEasy	EVs	210 ± 11	37.5 ± 15.1	3.00 ± 0.19	12.2 ± 4.3
	blank medium	200 ± 7	6.6 ± 0.2	0.06 ± 0.01	107.7 ± 1.7

Particle size and concentration were determined using scatter-based NTA, protein concentration was measured using the Micro BCA^™^ Protein Assay Reagent Kit. The results are presented as the mean ± standard deviation of two biological replicates. Particles to protein ratio was calculated for every biological replicate and presented as the mean ± standard deviation of two biological replicates. Results were normalized to the equivalent of EVs isolated from 1 × 10^6^ LPS-stimulated THP-1 cells as described in the method section. Equal volumes of blank cell culture medium, processed in parallel with the samples using UC or the exoEasy kit were included as controls to determine the background signals of both separation methods.

**Fig 3:**
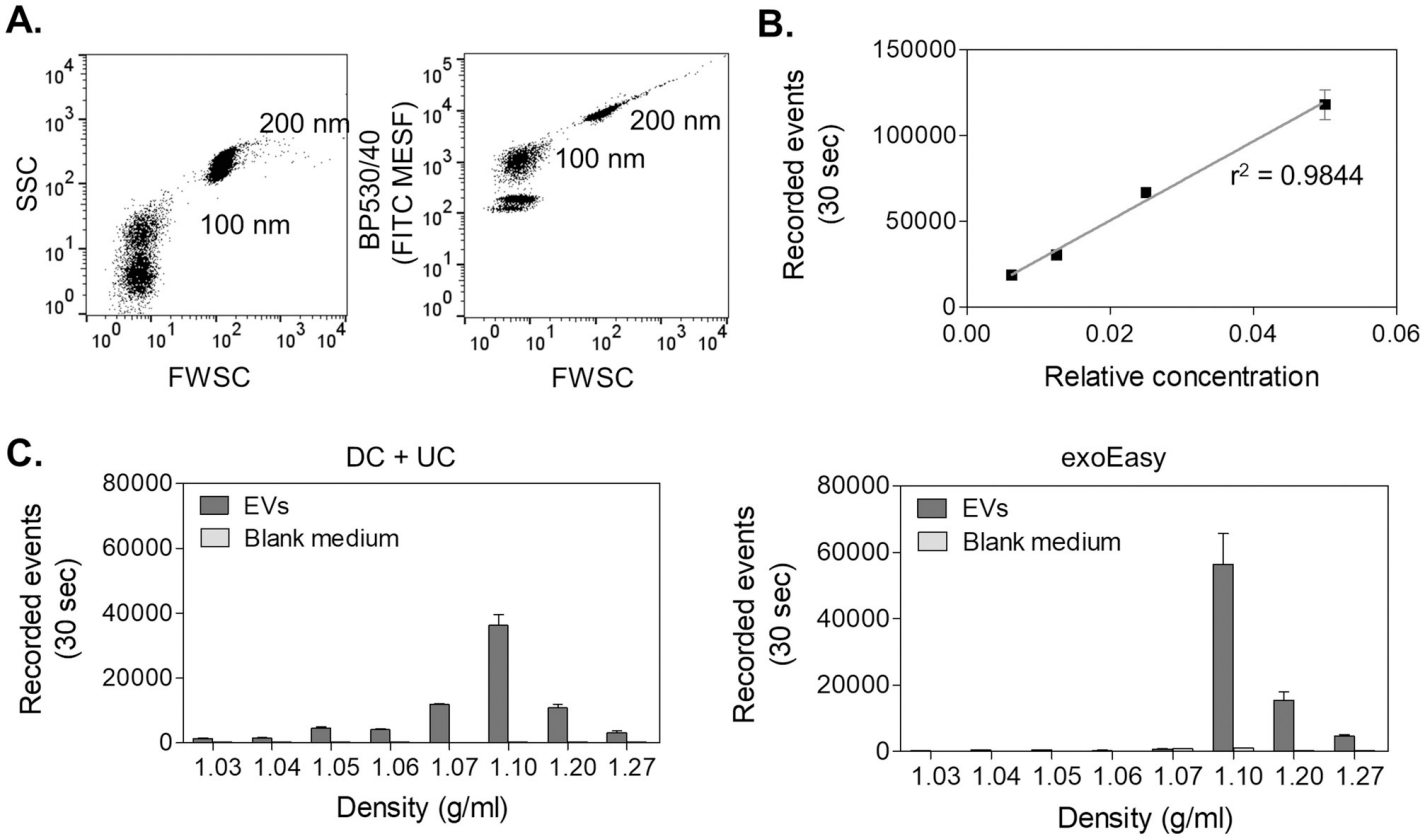
Characterization of EVs by HS-FCM after staining with CFDA-SE. **(A)** Dot plots of the side scatter (SSC) or fluorescence (BP530/40) expressed in units of FITC MESF *versus* forward scatter (FWSC) demonstrating the sensitivity of HS-FCM when measuring 100 and 200 nm fluorescent polystyrene beads. **(B)** Absence of detector swarming was verified using serially diluted CFDA-SE stained EVs from DC + UC isolated EVs. Additional fluorescence plots can be found in S6 Fig in S1 File. **(C)** A representative distribution of EV counts in different iodixanol density gradient fractions for both DC + UC (left) and exoEasy (right) isolated EVs and the corresponding blank medium controls. The results are presented as the average ± standard deviation of three technical replicates.

**Fig 4:**
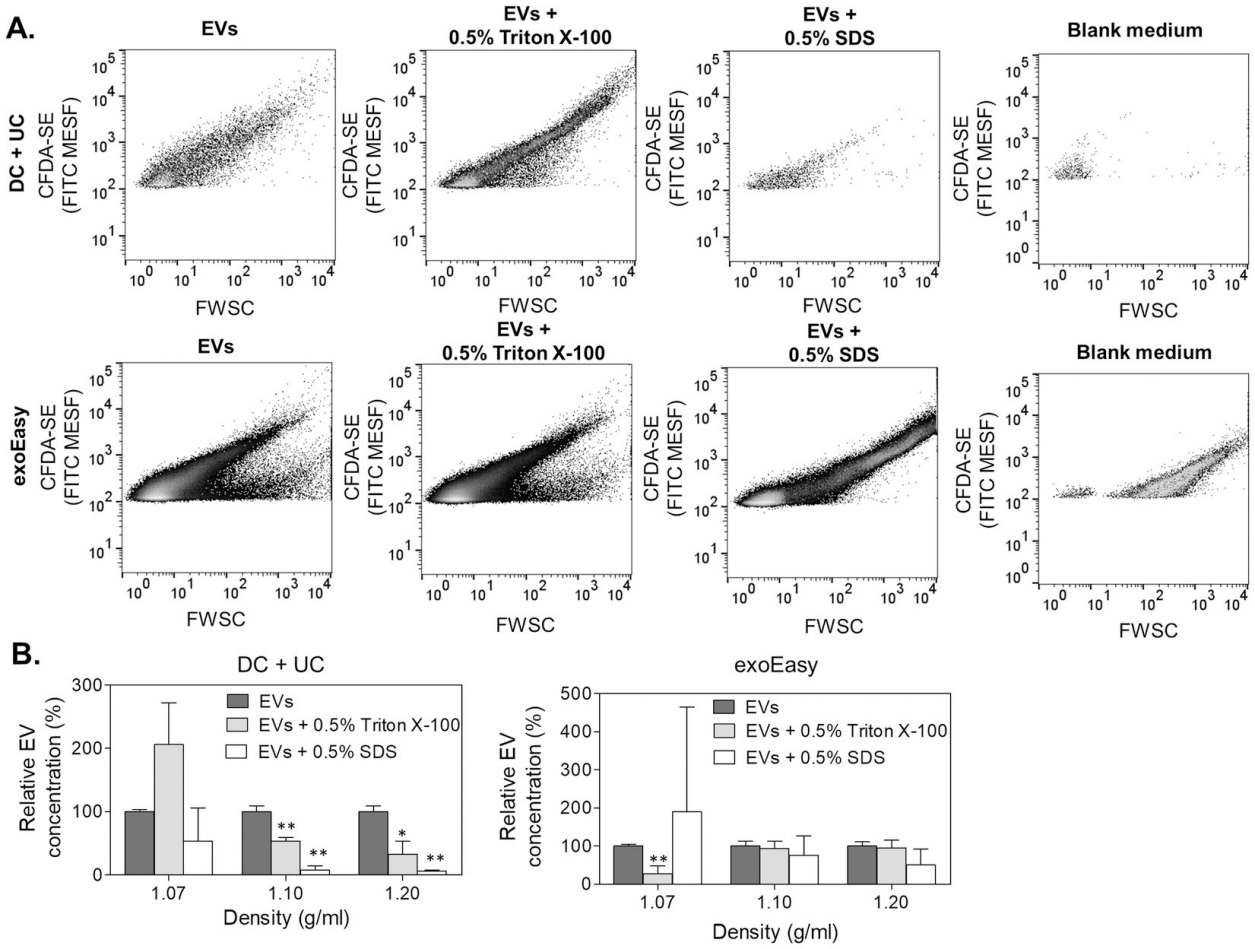
Evaluation of EV detergent sensitivity by HS-FCM after staining with CFDA-SE. **(A)** Dot plots of CFDA-SE fluorescence intensity expressed in units of FITC MESF as a function of forward scatter (FWSC) of EVs and blank medium controls with and without treatment with the detergents 0.5% (w/v) Triton X-100 or 0.5% (w/v) SDS. Representative examples of iodixanol fractions with a density of 1.10 g/ml are presented. Additional fluorescence plots of blank medium controls with and without treatment with the detergents can be found in S7 Fig in S1 File. **(B)** Relative EV numbers (%) measured during 30 seconds after detergent treatment compared to EVs without detergent (100%) in iodixanol fractions for DC + UC (left) and exoEasy (right) purified EVs. The results are presented as the average ± standard deviation of three biological replicates. For every biological replicate, the average was determined based on three technical replicate measurements. Statistically significant results are indicated by an asterisk (* p-value = 0.0031, ** p-value < 0.001).

Next, EVs were characterized by HS-FCM. Fluorescently labeled polystyrene beads of 100 and 200 nm were used to verify the instrument alignment (Fig 3A) and to validate repeatability among experiments (S5 Fig in S1 File). The freshly isolated EVs were stained with CFDA-SE. This dye permeates EV membranes by diffusion and is cleaved by intraluminal esterases to form the amine-reactive product carboxyfluorescein succinimidyl ester that produces a detectable fluorescence [36, 37] and serves as a fluorescent lumen-specific EV label [38]. Fluorescence-based triggering was used to identify the fluorescently stained EVs in the sample. In agreement with *Morales-Kastresana et al*. (2017) we observed that labeling of EVs with CFDA-SE increased the number of background noise events as a result of free dye which can hydrolyze spontaneously [37]. For that reason, it is crucial that the remainder of free dye is removed, and thus bottom-up density gradient centrifugation was utilized [24, 32]. In this purification step the fluorescently stained EVs will migrate to their buoyant density, while the free dye remains in the bottom fraction of the density gradient. EV concentrations were measured in the different iodixanol fractions for a fixed time interval of 30 seconds. False information on EV concentrations and scattering properties can arise when multiple particles are coinciding in the focus of the laser beam of the flow cytometer. In order to confirm the absence of this so-called “detector swarming” at the set sample flow rate, a dilution series of the stained EVs was prepared and measured (Fig 3B) [39]. The majority of the EVs were found in the iodixanol gradient fraction with a density of 1.10 g/ml, or at a higher density (Fig 3C). This agrees with other investigations which report typical EV density between 1.10 and 1.19 g/ml [15, 40]. Separation by DC + UC resulted in a broader density distribution of EVs in the different isolated fractions compared to the exoEasy kit, showing a peak at 1.10 g/ml, but also containing less dense EVs. DC + UC resulted in 49% of the total record events in the fraction corresponding to 1.10 g/ml, while for the exoEasy samples 72% of the total recorded events where found in this fraction. Further confirmation of the identity of EVs, as compared to protein aggregates and other contaminations, was obtained by analyzing the sensitivity to detergent treatment of the EVs in the different fractions [41–43]. For this, EVs were first stained with CFDA-SE for 1 hour, followed by treatment for 1 hour at room temperature with 0.5% (w/v%) Triton X-100 or 0.5% (w/v%) SDS combined with 30 minutes of sonication (Fig 4). Triton X-100 is one of the most commonly used non-ionic, non-denaturing aqueous detergents for solubilizing membrane proteins (critical micelle concentration (CMC) of 0.2 mM (0.0155% w/v) at 25°C), whereas SDS is an anionic surfactant which totally disrupts membranes and denatures proteins by breaking protein–protein interactions (CMC of 6 mM (0.17% w/v) at 25°C). Representative example plots of fluorescence intensity *versus* the forward scatter of CFDA-SE-labeled EVs before and after detergent treatment are shown for the iodixanol fraction with a density of 1.10 g/ml (Fig 4A). The forward scatter (FWSC) is indicative of particle size, but is also affected by a particle’s refractive index and its shape [21, 44]. The population of recorded entities isolated with DC + UC was different compared to the events obtained by exoEasy. More particles with low FWSC were present in the exoEasy samples. An additional population with high FWSC and low CFDA-SE fluorescence intensity was identified, which was also present in the blank medium, which could be a sign for the presence of protein aggregates or other co-isolating compounds in the exoEasy processed samples. Treatment with 0.5% (w/v) Triton X-100 did not destabilize EVs in exoEasy isolated samples, whereas for DC + UC samples the EV distribution shifted towards lower FWSC, indicating smaller vesicle sizes and/or reduced refractive index and/or altered shape. In contrast, SDS treatment resulted in a statistically significant reduction in EV numbers for DC + UC, but not for the exoEasy samples. In the latter, however, a shift towards an increased number of EVs with higher CFDA-SE staining intensity and higher FWSC was observed, suggesting an increase in EV size or EV agglomeration and/or increased EV refractive index. Remarkably, the blank medium-derived population disappeared by SDS treatment. This was also observed by NTA when the elution buffer after processing of the exoEasy kit was exposed to 0.5% SDS (S2B Fig in S1 File), indicating that these background events are sensitive to anionic surfactants. In the EV iodixanol fractions with a density of 1.20 g/ml similar observations as for 1.10 g/ml were found (Fig 4B). However, in fractions with a density of 1.07 g/ml, DC + UC isolates showed an increase in relative EV numbers in the presence of Triton X-100, while SDS did not show a statistically significant difference compared to untreated EVs. This was opposite to EVs isolated using exoEasy, which showed a statistically significant decrease in EV number in the presence of Triton X-100, but (non-significantly) increased EV counts when SDS was added.

Taken together, these results suggest that the DC + UC centrifugation and exoEasy kit resulted in isolates of EVs with a different composition and purity. Both scatter-based NTA and fluorescence-triggered HS-FCM analyses showed a noteworthy amount of large particles in the exoEasy blank medium samples, which is in line with previous investigations suggesting that the observed extra particles could be attributed to flow-through of the kit elution buffer (S2 Fig in S1 File) [10]. Those extra particles could have a substantial degree of autofluorescence and therefore be detected in fluorescence-triggered HS-FCM. Sensitivity to detergents has been proposed as a method to confirm the vesicular nature of the isolated compounds [10]. Here, EVs isolated with the exoEasy kit were largely insensitive to detergent treatment, while DC + UC isolated EVs were completely destroyed with 0.5% (w/v) SDS. This suggests that an important part of the particles detected in exoEasy isolates are not EVs. Western blot analysis, however, showed stronger enrichment of EV markers in the exoEasy compared to DC + UC isolated samples. The presence of those markers could potentially also come from contaminating cell debris, which agrees with the presence of mitochondrial components indicated by the presence of cytochrome c [45]. Alternatively, that exoEasy results in the separation of more EVs, but it is possible that due to EV agglomeration those vesicles are more difficult to permeabilize compared to single EVs isolated using DC + UC.

### EV storage at 4°C or -80°C does not affect their concentration and intactness

**Fig 5:**
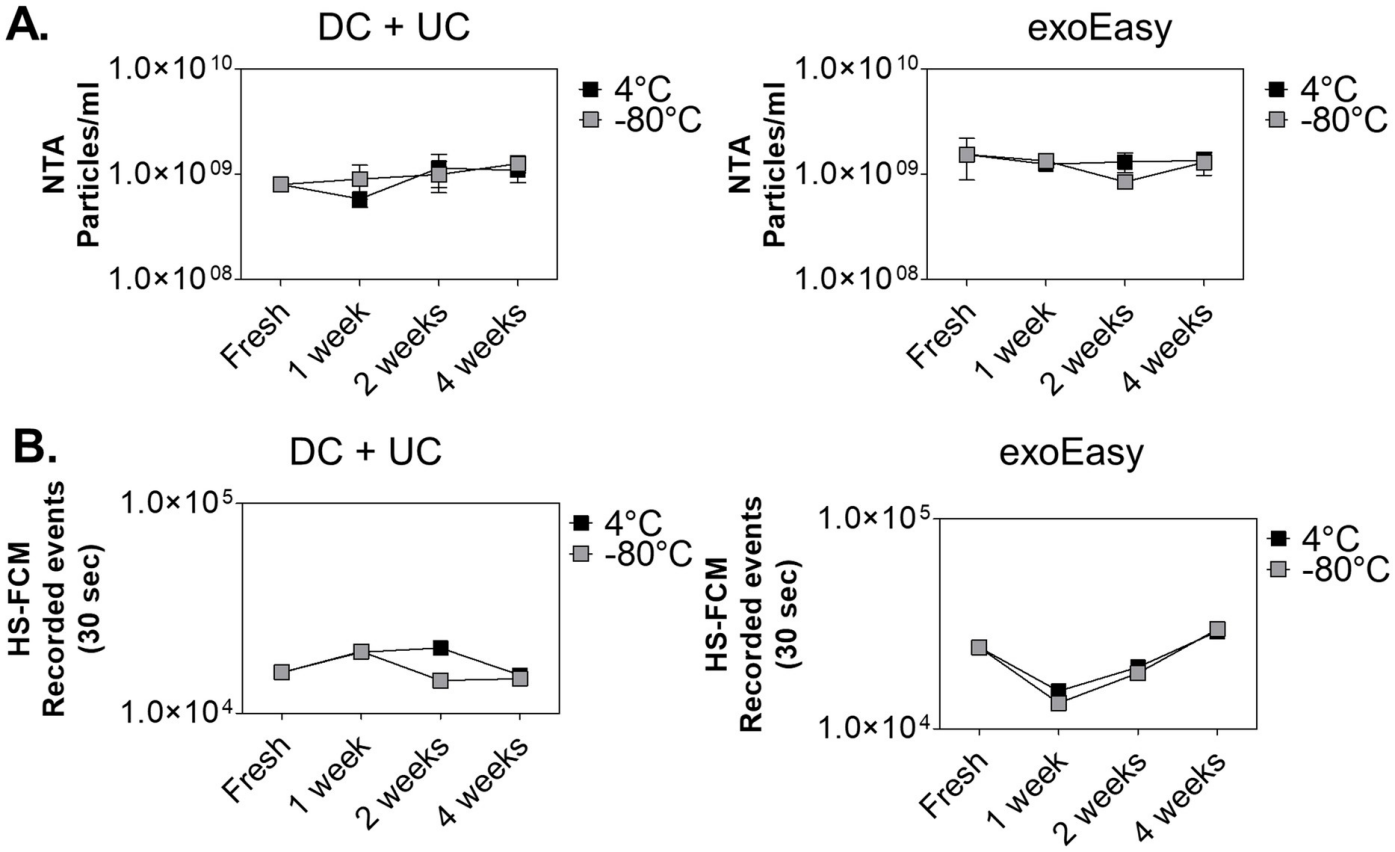
Effect of storage at 4°C or -80°C on EV concentrations. EV number concentrations were evaluated using **(A)** scatter-based NTA and **(B)** fluorescence-triggered HS-FCM on fresh isolates obtained using DC + UC or exoEasy, and after 1 week, 2 weeks and 1 month of storage. A representative experiment out of two is shown. The NTA concentrations were determined in triplicate and results are shown as the average ± standard deviation. For the HS-FCM measurements, the total number of particles in 30 seconds was determined.

In order to evaluate the effect of storage on EV number concentrations, EVs were first separated from conditioned medium of LPS-stimulated THP-1 cell culture by DC + UC or exoEasy. Thereafter, samples were aliquoted, stored at 4°C or -80°C in PBS (DC + UC method) or elution buffer (exoEasy kit). At different time intervals samples were thawed for subsequent measurement using scatter-based NTA and HS-FCM after labeling with CFDA-SE, followed by bottom-up density gradient purification (Fig 5). When comparing preservation at 4°C or -80°C after 1 week, 2 weeks and 1 month with freshly isolated samples, no differences in EV number concentrations were found. Surprisingly, HS-FCM analysis of EVs stored at 4°C resulted in similar EV concentrations compared to the vesicles stored at -80°C, indicating that they remain intact under these low temperature conditions as the intravesicular enzymes needed for the fluorescence activation of the CFDA-SE dye remain stable in the medium long term. *Geeurickx et al* demonstrated that EVs were stable at 4°C for at least 1 week [28], which is in agreement with our results. Additionally, similar to our findings they did not observe interference of storage at -80°C on EV concentration and EV intactness, which is also supported by other studies [46, 47]. Although no interference of EV storage was found on particle number and intactness, our data does not provide evidence on prolonged stability of EV cargo and functionality. More research is needed to further address those questions.

### Fluorescence intensity of labelled EVs is stable at -80°C, but not at 4°C

**Fig 6:**
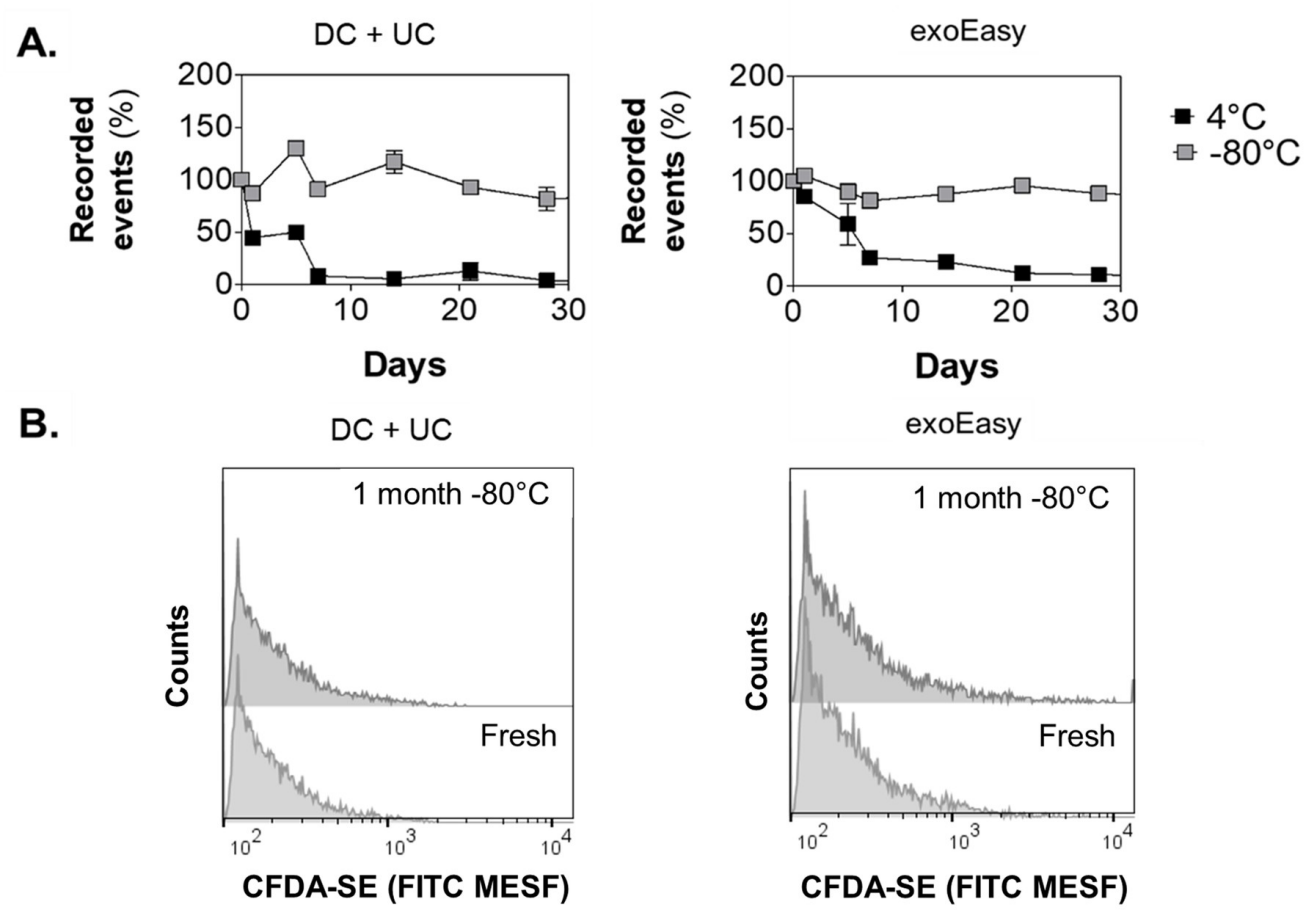
Effect of storage on stability of EV staining fluorescence determined by HS-FCM. **(A)** Samples were labeled with CFDA-SE, purified on a density gradient and EV counts (density of 1.10 g/ml) measured for 30 seconds. Relative number of recorded events (%) in 30 seconds measured after different times up to 28 days of storage at 4°C or -80°C in sucrose. Normalization of events was performed between independent experiments (n = 4) and are presented as the average percentage of recorded events compared to the number of events at day 0 with error bars indicating the standard error of the mean. **(B)** A representative number distribution of the fluorescence intensity (expressed in units of FITC MESF) after one month of storage at -80°C shows that the intensity distribution is comparable between fresh and stored samples. When samples were stored at 4°C, hardly any fluorescent events were found (not shown).

The stability of the intravesicular fluorescence intensity acquired by labeling of EVs using CFDA-SE was also evaluated over a period of one month. For this, fresh EV samples were stained with CFDA-SE, purified using bottom-up density gradient centrifugation and measured using HS-FCM. Technical replicate aliquots of the density fractions containing the labelled EVs were individually stored at 4°C or -80°C. At subsequent time intervals, labelled EVs were thawed and left to equilibrate at room temperature and measured by HS-FCM (Fig 6). While samples obtained by the two isolation methods and stored at -80°C remained stable over time, samples stored at 4°C rapidly lost their fluorescence intensity from the first day of storage onward. These findings are in agreement with *Lőrincz et al*, in which the number of stained EVs derived from neutrophilic granulocytes was already decreased after 1 week and significantly decreased after 1 month of storage at 4°C [20]. As unstained EV fractions stored at 4°C and -80°C have shown to remain equally stable, this observation of loss of fluorescence intensity upon storage of CFDA-SE-labeled EVs is probably due to instability of the dye itself when samples were stored at 4°C, rather than unstable EVs.

## Conclusion

In-depth characterization of EVs is an important parameter for the standardization and the comparison of separation methods of EVs from biofluids. In this work, we have demonstrated the power of combining different characterization techniques with HS-FCM to elucidate differences in EV composition, yield and purity between the DC + UC and affinity-based exoEasy separation methods. It has furthermore been shown that inclusion of a minimum of control measurements (such as medium background, EV lysis by detergent, removal of free CFDA-SE from labeled EVs) is crucial to differentiate between EVs and co-isolated or process-related contaminants. Moreover, we have shown that the storage of EVs at 4°C and -80°C up to one month is suited for further downstream quantification of EV concentrations. For preservation of CFDA-SE-labeled EVs on the other hand, our data indicate preferential storage at -80°C.

## Supporting information

S1 File Supporting information containing S1-S7. (PDF)

S1 Raw (PDF)
