# Supplementary material for: Comparison of extracellular vesicle isolation and storage methods using high-sensitivity flow cytometry
Source: PLoS One. 2021 Feb 4;16(2):e0245835. doi: 10.1371/journal.pone.0245835 (PMC7861365; doi:10.1371/journal.pone.0245835)
Supplement: S1 File — (PDF) [file pone.0245835.s001.pdf]

Supporting Information

Comparison of extracellular vesicle isolation and storage methods using high-sensitivity flow cytometry

Sarah Deville[1,2], Pascale Berckmans[1], Rebekka Van Hoof[1,3,4], Ivo Lambrichts[2], Anna Salvati[5], Inge Nelissen[1*]

[1] Health Unit, Flemish Institute for Technological Research, Mol, Belgium

[2] Biomedical Research Institute, Hasselt University, Diepenbeek, Belgium

[3] Theoretical Physics, Hasselt University, Diepenbeek, Belgium

[4] Laboratory for Soft Matter and Biophysics, KU Leuven, Leuven, Belgium

[5] Groningen Research Institute of Pharmacy, University of Groningen, Groningen, The Netherlands

* Corresponding author

E-mail: inge.nelissen@vito.be

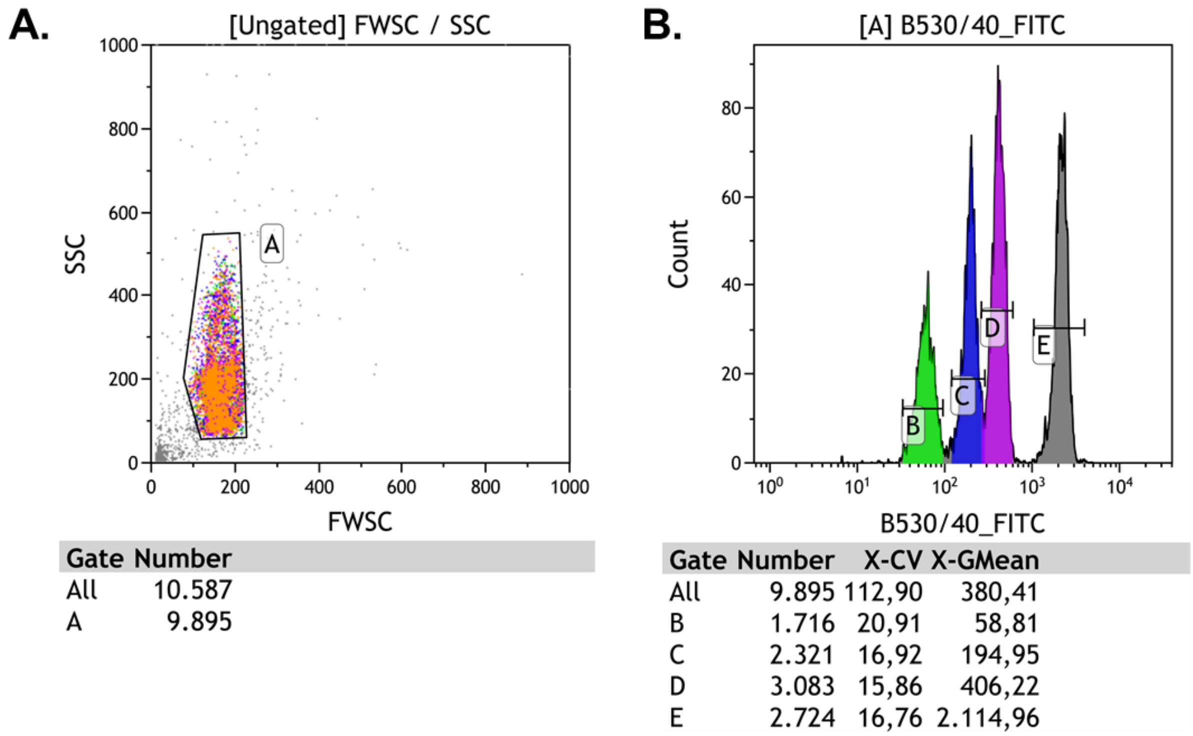

Supplementary Fig 1. Flow cytometry of FITC MESF Beads. (A) Side scatter (SSC) is plotted against forward scatter (FWSC). A gate `A` was set around the positive population. **(B)** The number of positive events from population A defined by the gate is plotted against the FITC fluorescence intensity. For each peak (B-E), the geomean was calculated using Kaluza Analysis Software, version 2.1 (Beckman Coulter).

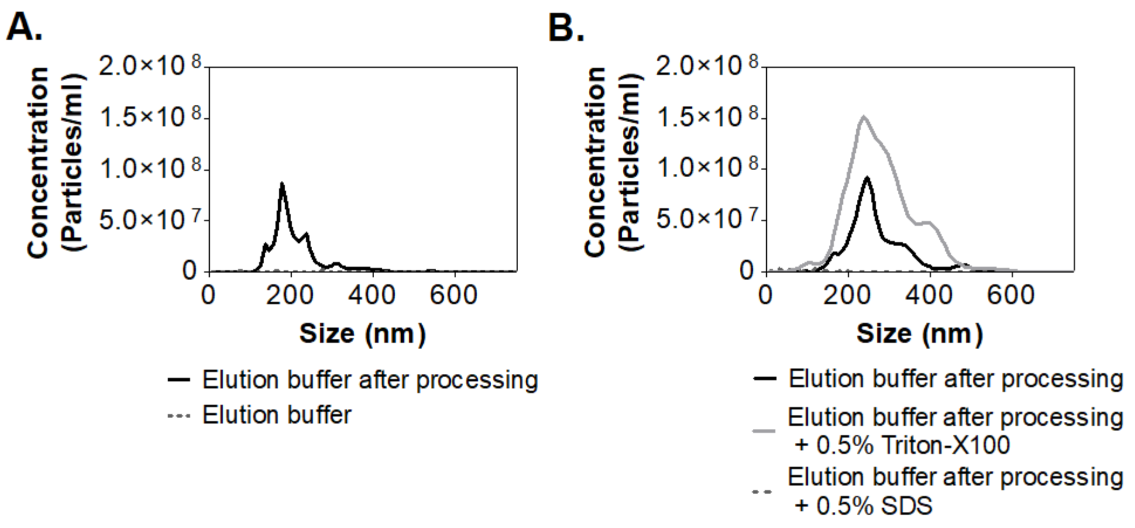

Supplementary Fig 2. Size distribution of the exoEasy background when performing scatter-based NTA. (A) A representative distribution profile is shown of the elution buffer before (dashed line, this line mainly coincides with the x-axis) and after processing through the exoEasy column (solid line) using the manufacturer's procedure. **(B)** A representative distribution profile of the elution buffer after processing through the exoEasy column (black solid line) incubated at room temperature for 1 hour together with the addition of 0.5% Triton-X100 or 0.5% SDS combined with 30 minutes of sonication.

A.

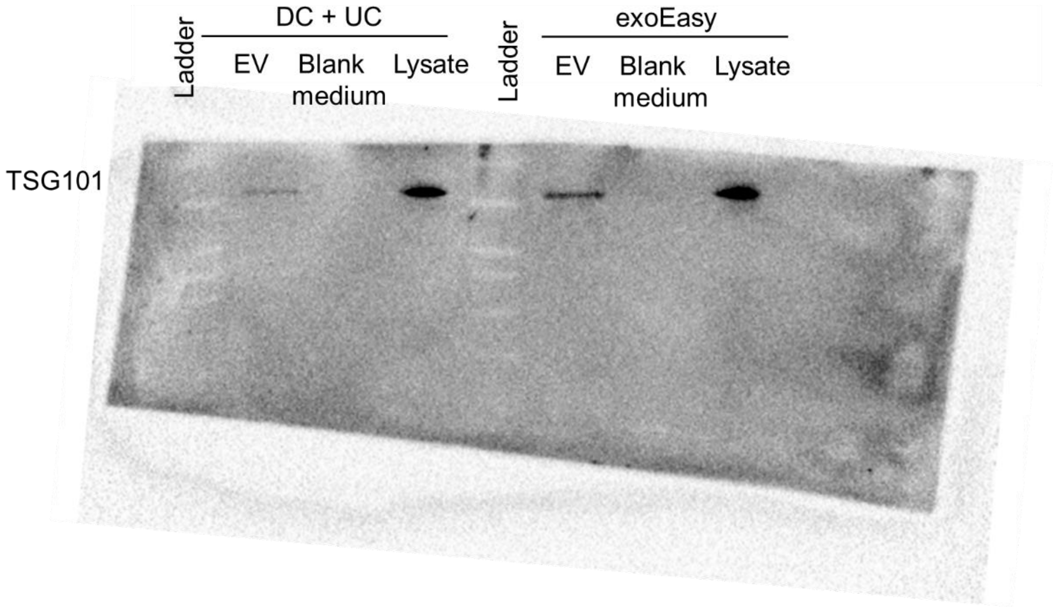
Ladder
DC + UC
EV
Blank medium
Lysate
Ladder
exoEasy
EV
Blank medium
Lysate
TSG101

B.

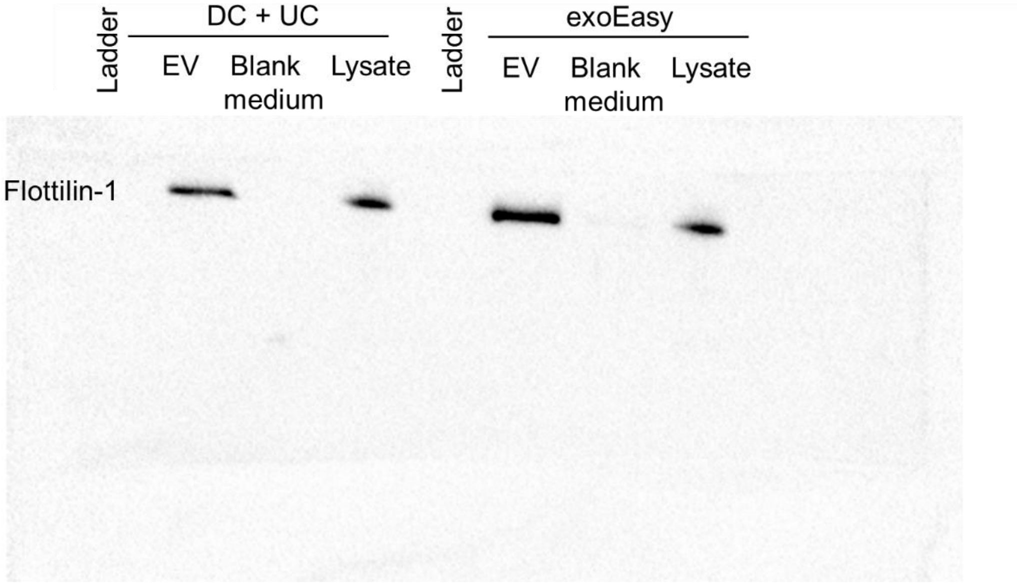
Ladder
DC + UC
EV
Blank medium
Lysate
Ladder
exoEasy
EV
Blank medium
Lysate
Flottilin-1

C.

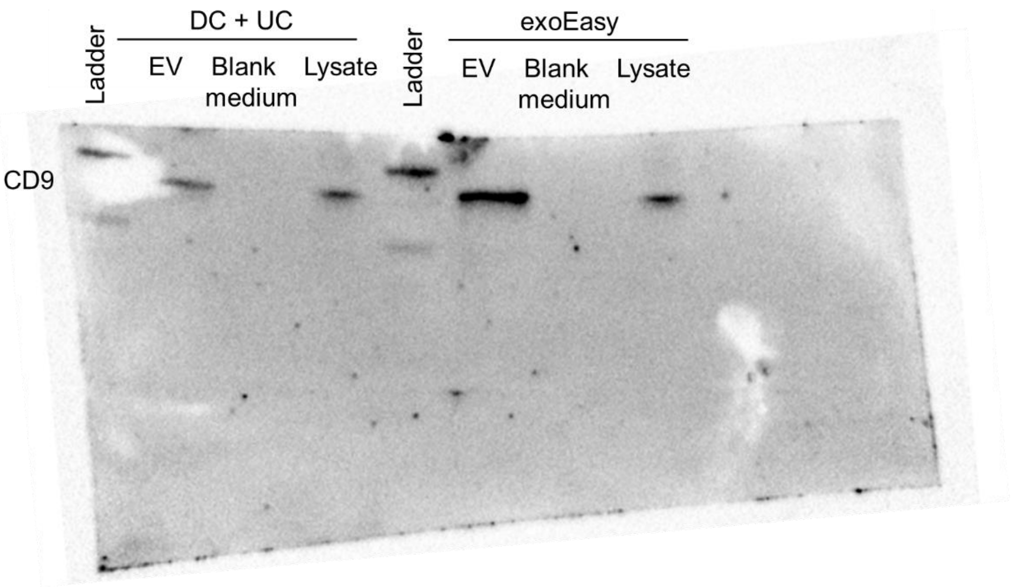

D.

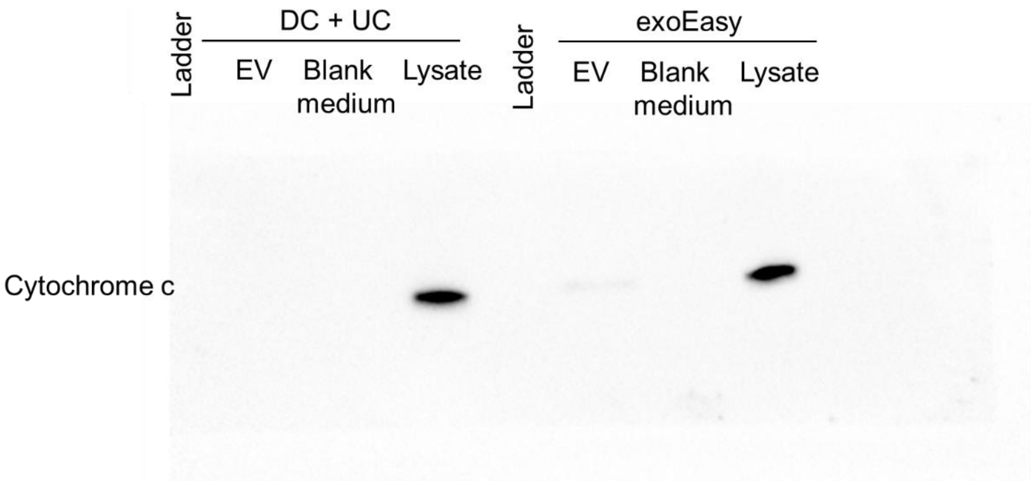

Supplementary Fig 3. Western blotting analysis of EVs. Separate immunoblots for the detection of proteins considered as EV-specific markers **(A)** TSG101, **(B)** flottilin-1 and **(C)** CD9 and the non-EV marker **(D)** cytochrome c for both separation procedures. The presence or absence of the protein markers in isolated EV fractions was evaluated against blank medium (negative control) and LPS-stimulated THP-1 cell lysate (positive control). The raw data is accessible using FigShare.com.

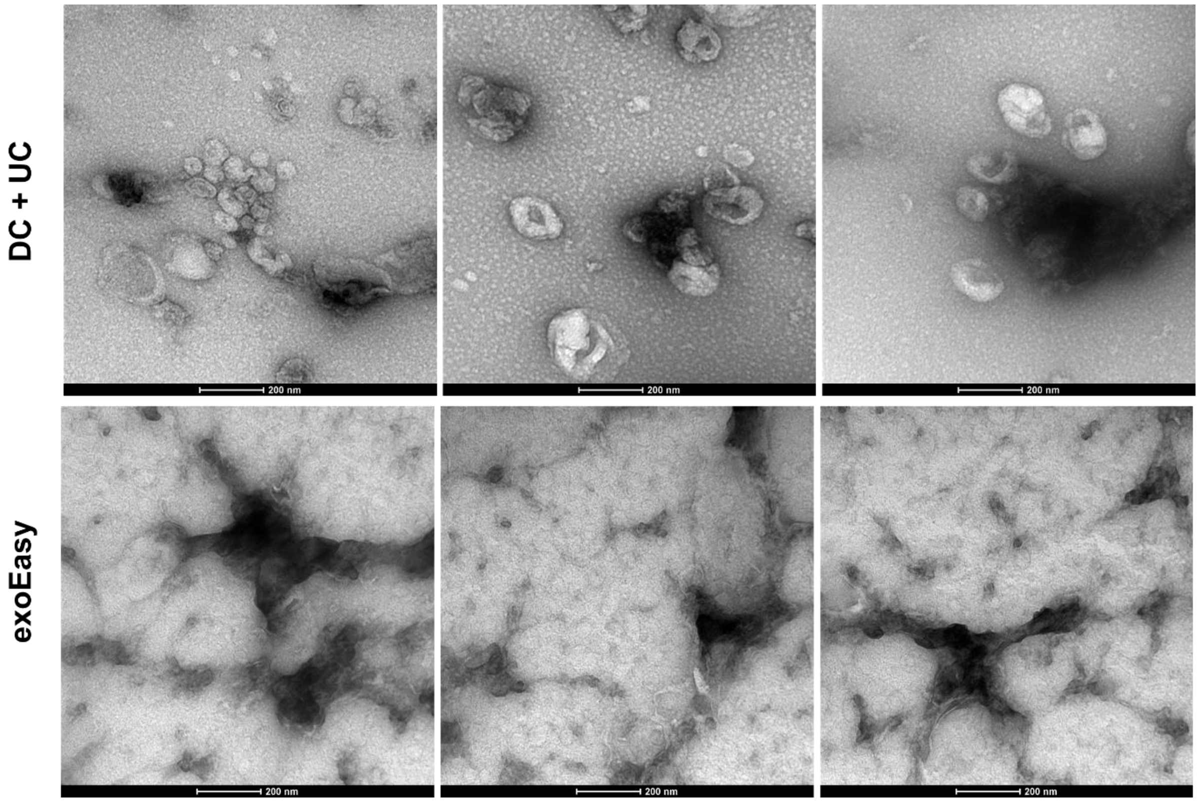

Supplementary Fig 4. Electron microscopy of EVs isolated using DC + UC and exoEasy purification Three representative images are shown. Scale bar: 200 nm

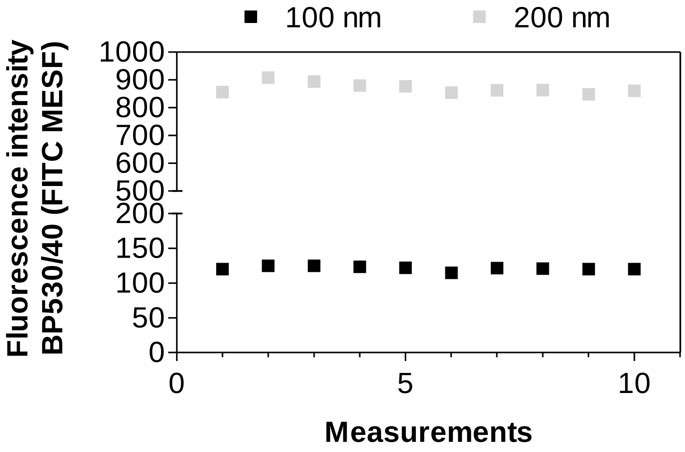

Supplementary Fig 5. Repeatability of HS-FCM measurements. Measurement of 100 and 200 nm fluorescent polystyrene beads over multiple experimental days. The fluorescence intensity values are expressed in units of FITC MESF. Fresh dilutions of the beads were prepared, and samples were measured before acquisition of the EV samples.

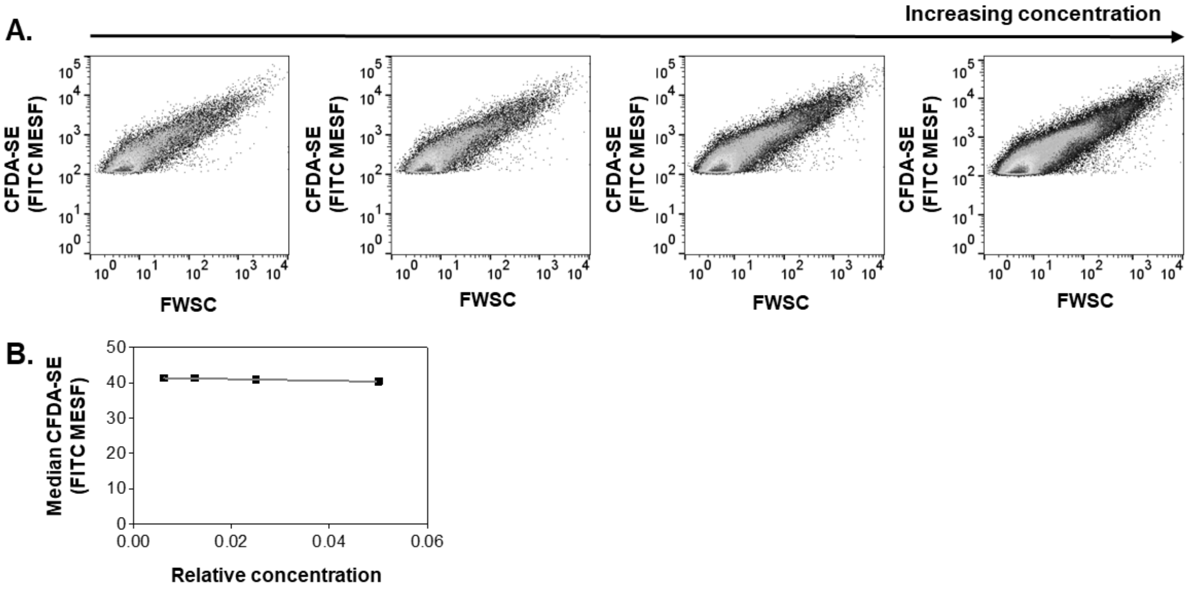

Supplementary Fig 6. Absence of detector swarming was verified using serially diluted CFDA-SE stained EVs from DC + UC isolated EVs. **(A)** Additional plots from Fig 3B are shown. **(B)** The median CFDA-SE intensity (expressed in units of FITC MESF) over the different dilutions remains stable and equals roughly 41 units of FITC MESF.

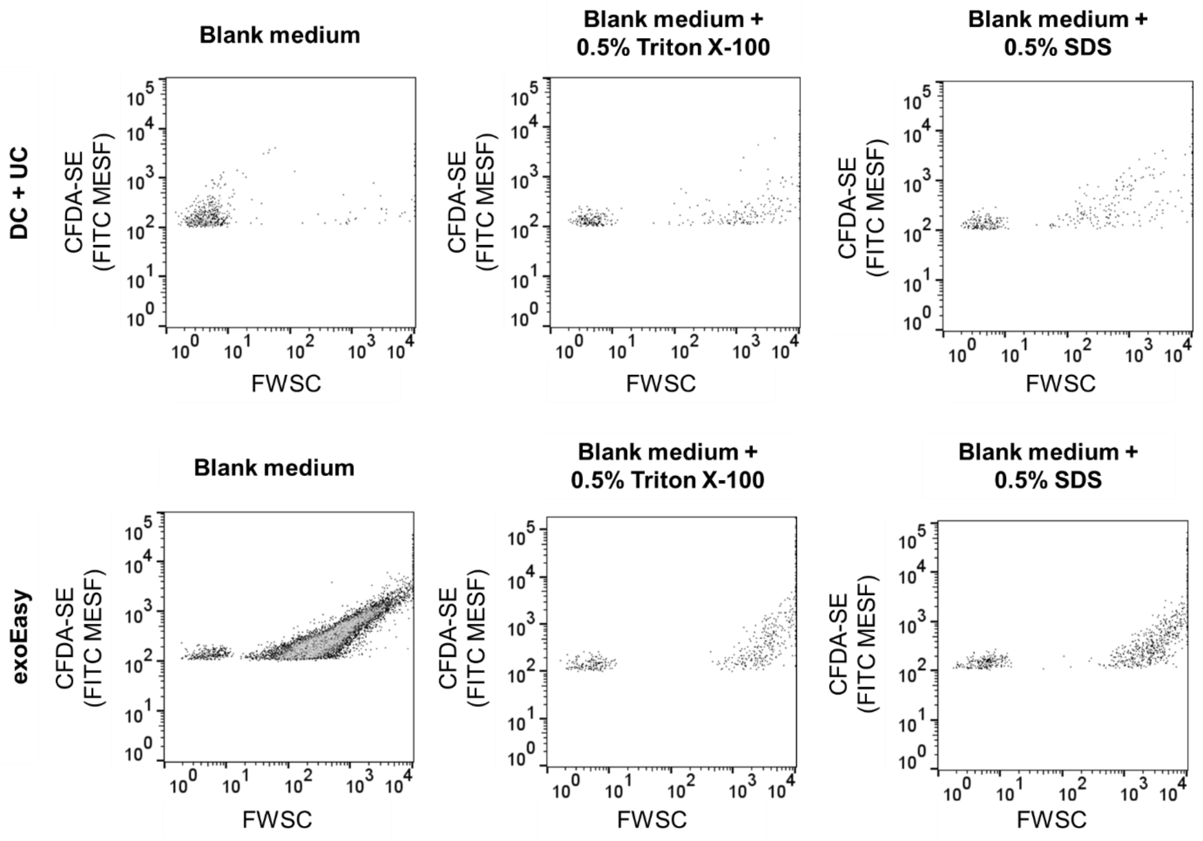

Supplementary Fig 7. Evaluation of interaction of blank medium and detergents by HS-FCM after staining with CFDA-SE. Dot plots of CFDA-SE fluorescence intensity (expressed in units of FITC MESF) as a function of forward scatter (FWSC) of blank medium with and without treatment with the detergents 0.5% (w/v) Triton X-100 or 0.5% (w/v) SDS. Representative examples of iodixanol fractions with a density of 1.10 g/ml are presented.
