# Supplementary material for: Comparison of extracellular vesicle isolation and storage methods using high-sensitivity flow cytometry
Source: PLoS One. 2021 Feb 4;16(2):e0245835. doi: 10.1371/journal.pone.0245835 (PMC7861365; doi:10.1371/journal.pone.0245835)
Supplement: S1 Raw — (PDF) [file pone.0245835.s002.pdf]

TSG101

Molecular weight: 44 kDa

Fig 2C

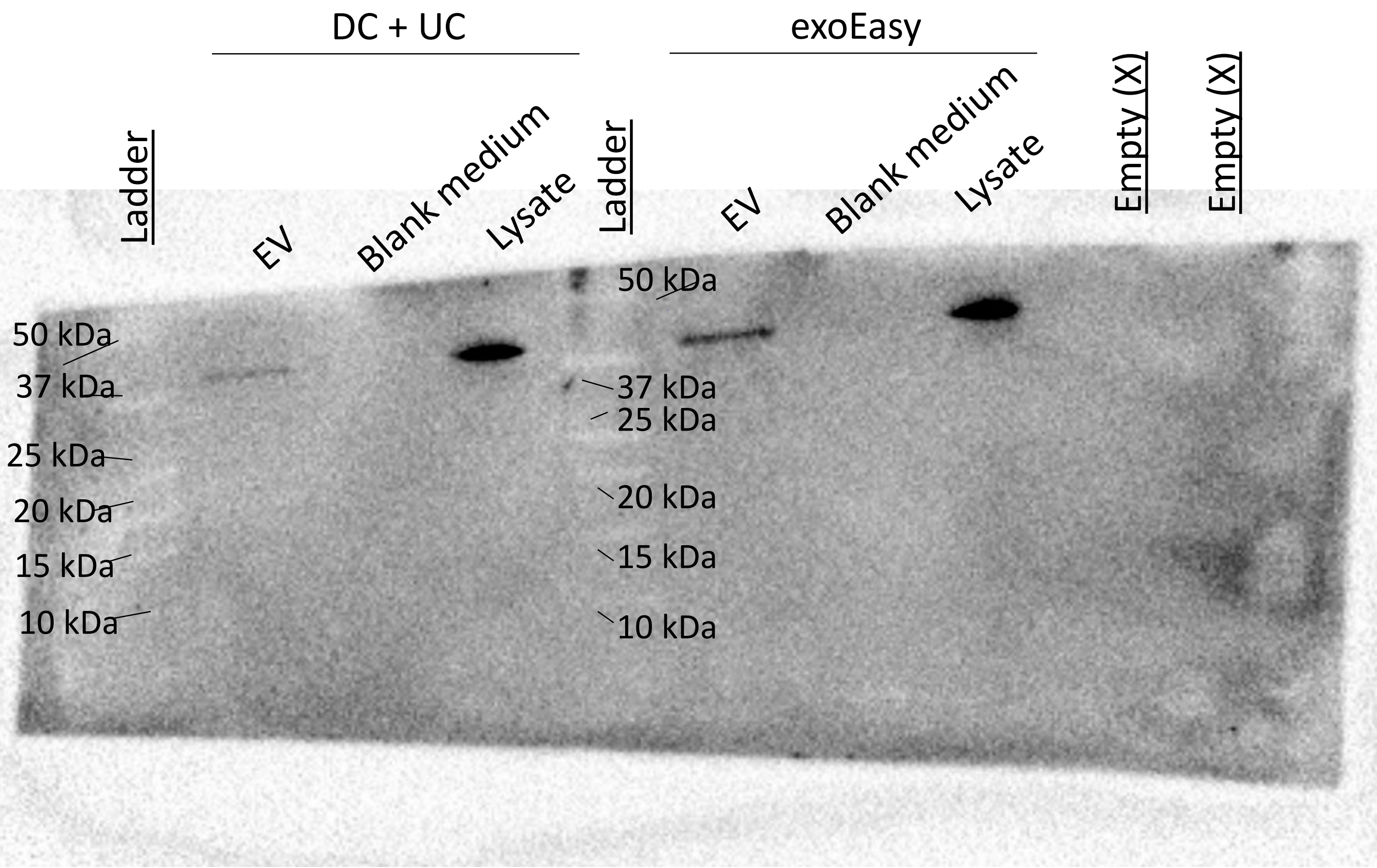

Image Details | Analysis Settings | Notes

Acquisition Information

Imager	ChemiDoc XRS+
Exposure Time (sec)	5.000 (Manual)
Flat Field	Applied (Lens)
Serial Number	721BR11342
Software Version	5.1
Application	Chemi
Excitation Source	No Illumination
Emission Filter	No Filter
Binning	3x3

Image Details | Analysis Settings | Notes

No analysis performed

Flottilin-1

Molecular weight: 47 kDa

Fig 2C

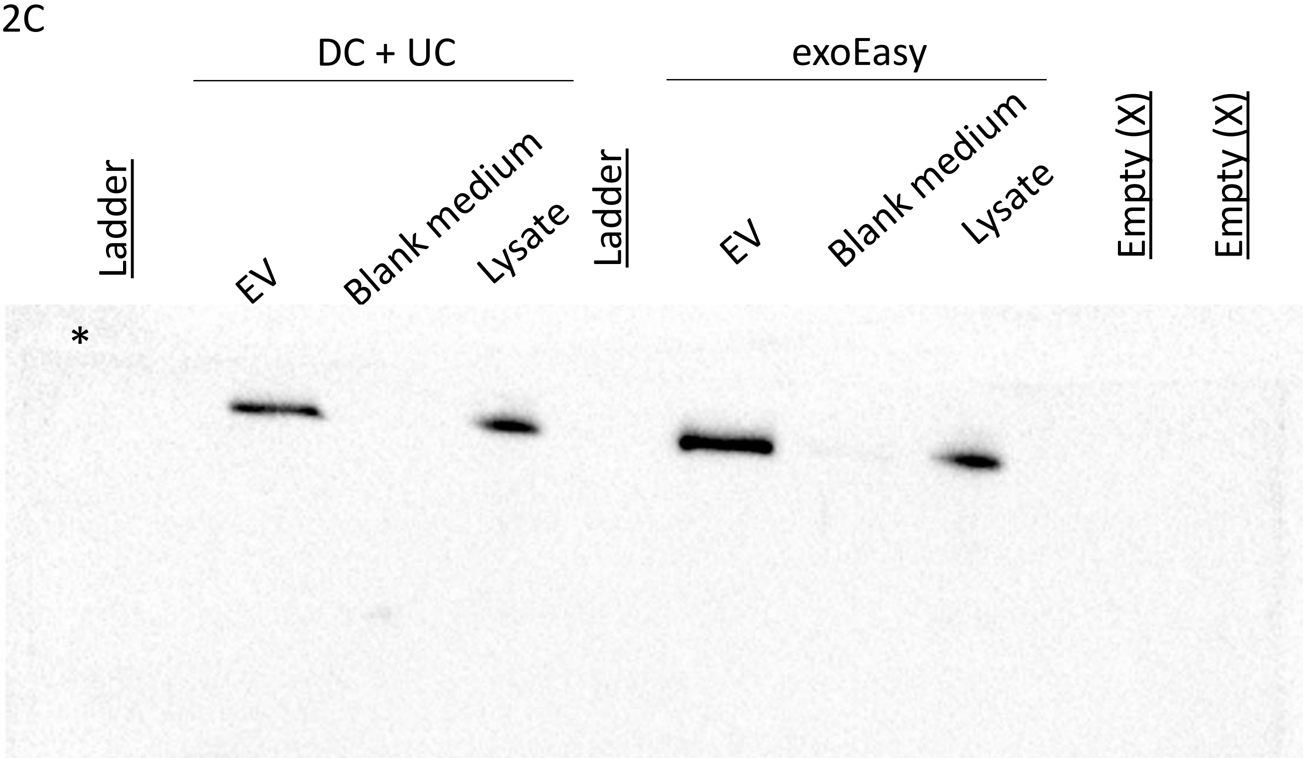

Ladder (brightfield):

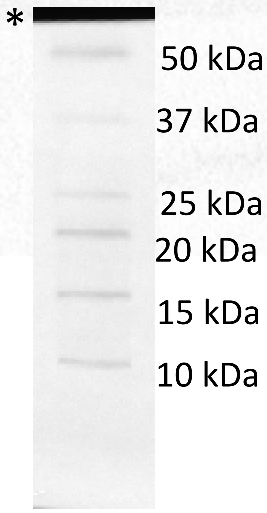

Ladder was visualized using white light and epi illumination

The asterix (*) corresponds with the upper part of the gel where the ladder is corresponding too. The molecular weight bands of flottilin-1 are localized between 50 and 37 kDa.

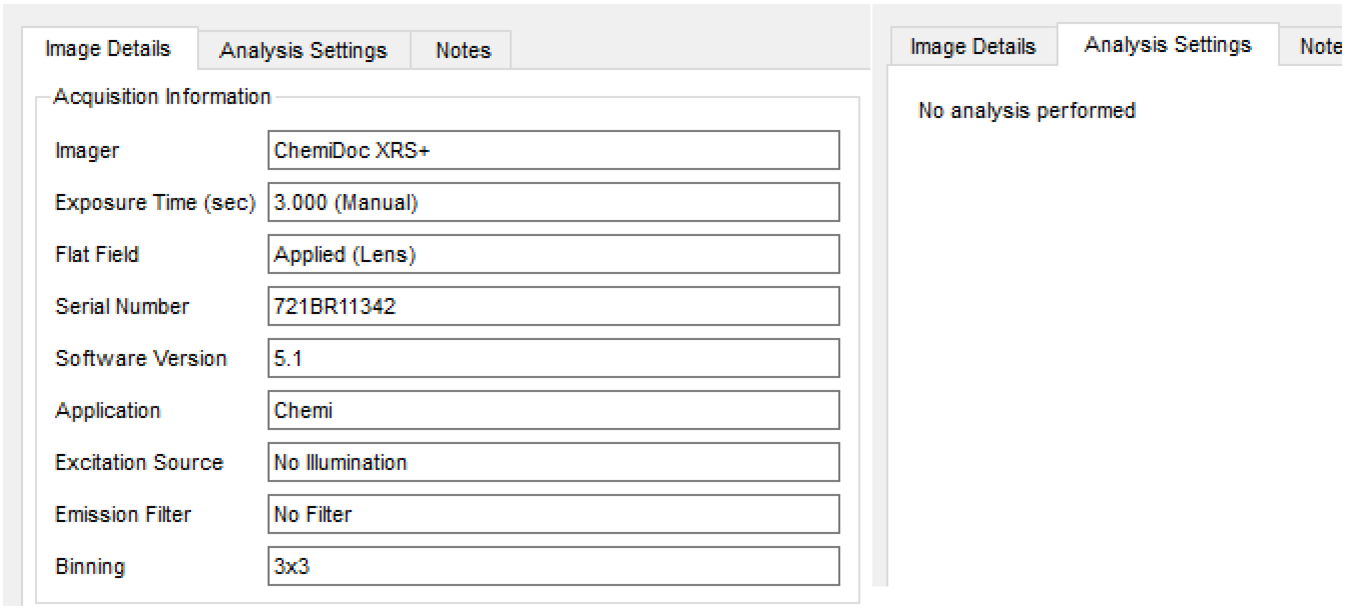

Image Details | Analysis Settings | Notes

Acquisition Information

Imager	ChemiDoc XRS+
Exposure Time (sec)	3.000 (Manual)
Flat Field	Applied (Lens)
Serial Number	721BR11342
Software Version	5.1
Application	Chemi
Excitation Source	No Illumination
Emission Filter	No Filter
Binning	3x3

Image Details | Analysis Settings | Note

No analysis performed

CD9

Molecular weight: 24-27 kDa

Fig 2C

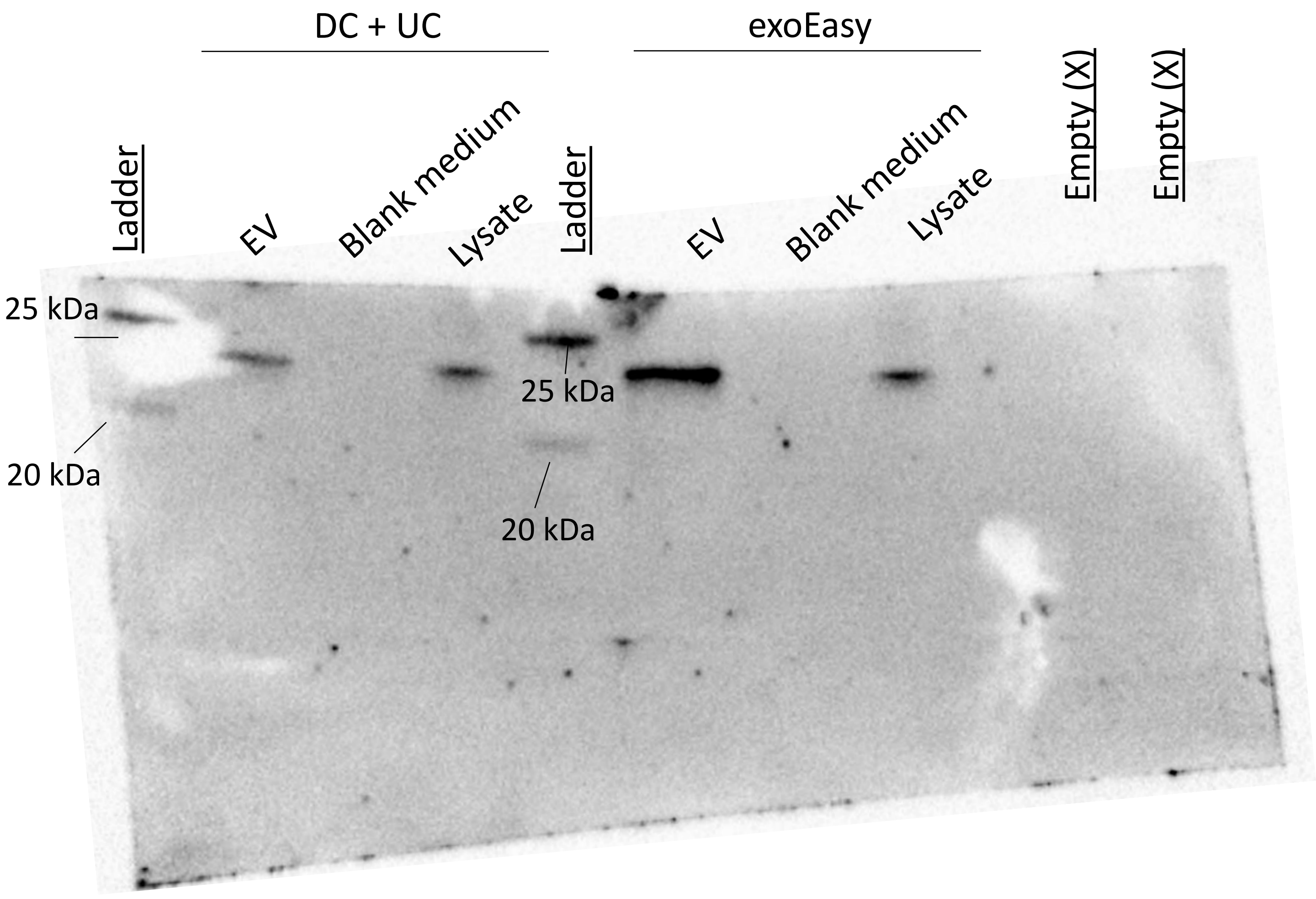

Image Details | Analysis Settings | Notes

Acquisition Information

Imager	ChemiDoc XRS+
Exposure Time (sec)	2.000 (Manual)
Flat Field	Applied (Lens)
Serial Number	721BR11342
Software Version	5.1
Application	Chemi
Excitation Source	No Illumination
Emission Filter	No Filter
Binning	3x3

Image Details | Analysis Settings | Notes

No analysis performed

Cytochrome c

Molecular weight: 12 kDa

Fig 2C

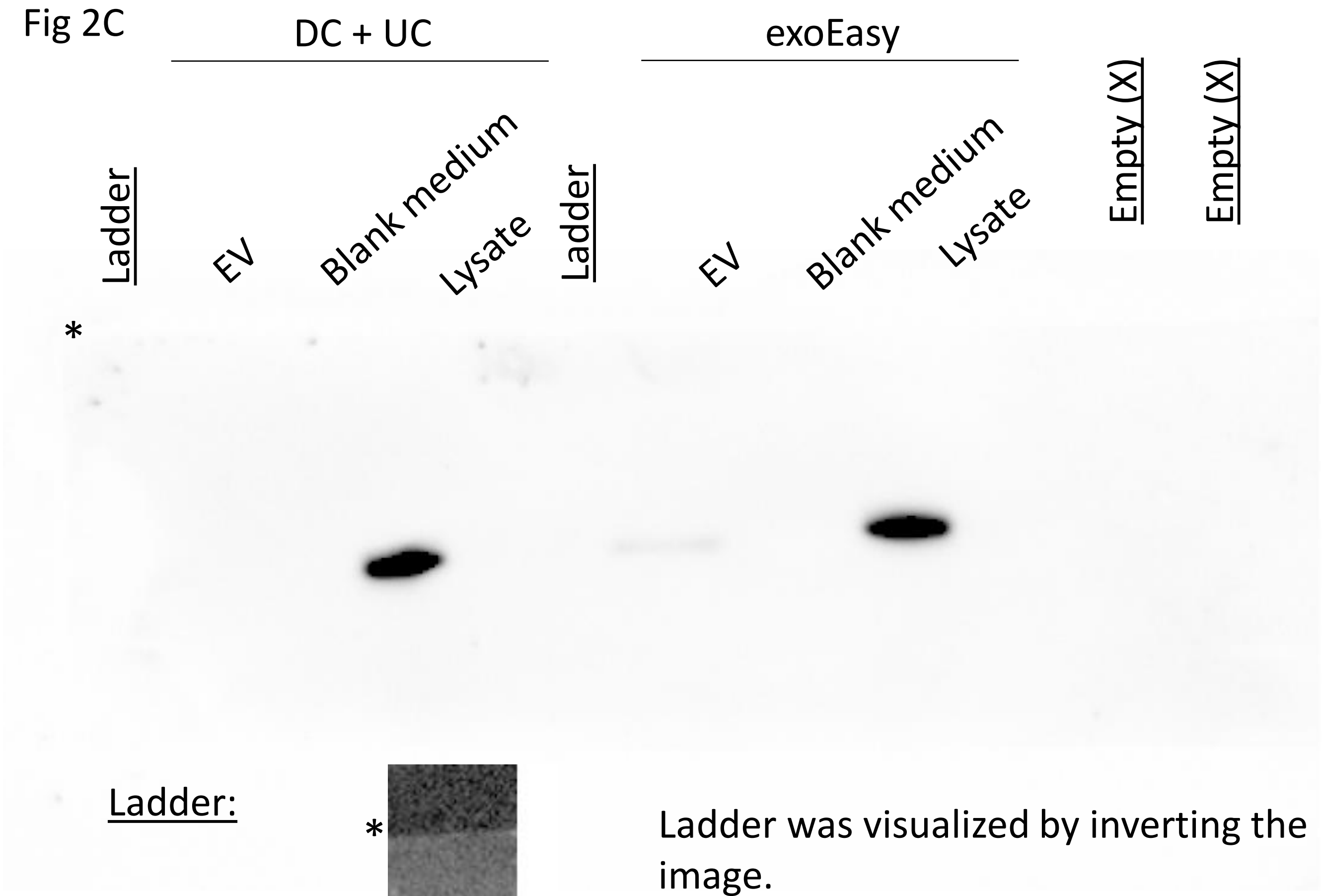

Ladder:

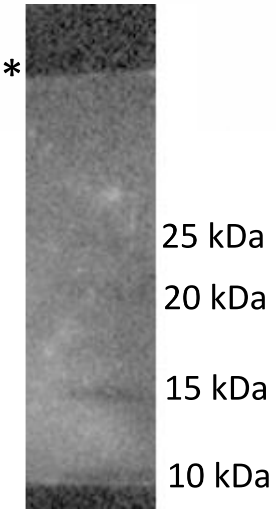

Ladder was visualized by inverting the image.
The asterix (*) corresponds with the upper part of the gel where the ladder is corresponding too. The molecular weight bands of cytochrome c are localized between 20 and 15 kDa.

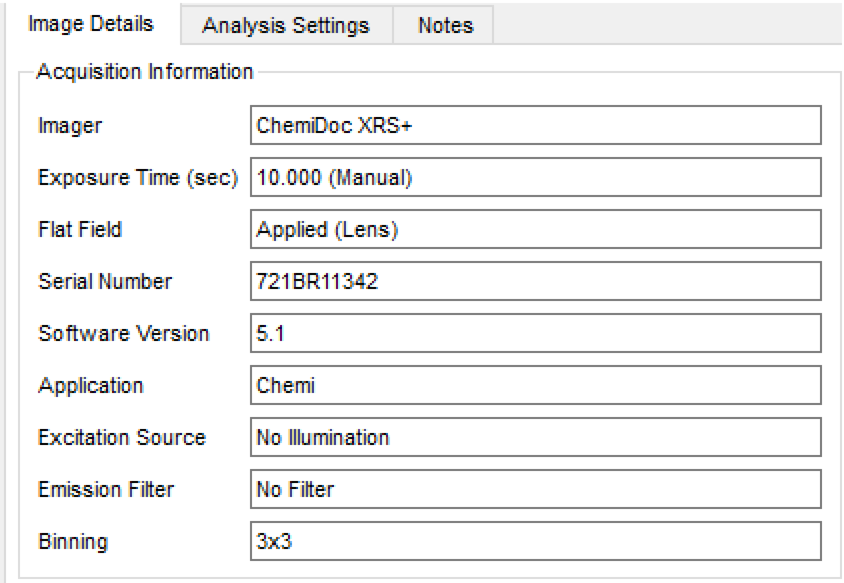

Image Details | Analysis Settings | Notes

Acquisition Information

Imager	ChemiDoc XRS+
Exposure Time (sec)	10.000 (Manual)
Flat Field	Applied (Lens)
Serial Number	721BR11342
Software Version	5.1
Application	Chemi
Excitation Source	No Illumination
Emission Filter	No Filter
Binning	3x3

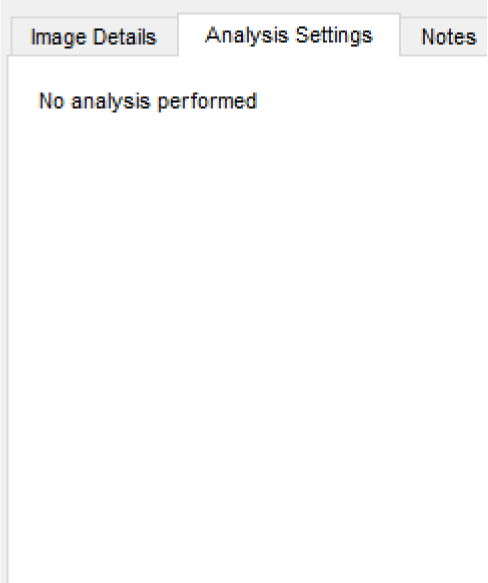

Image Details | Analysis Settings | Notes

No analysis performed
